# Microbiome and infectivity studies reveal complex polyspecies tree disease in Acute Oak Decline

**DOI:** 10.1038/ismej.2017.170

**Published:** 2017-10-13

**Authors:** Sandra Denman, James Doonan, Emma Ransom-Jones, Martin Broberg, Sarah Plummer, Susan Kirk, Kelly Scarlett, Andrew R Griffiths, Maciej Kaczmarek, Jack Forster, Andrew Peace, Peter N Golyshin, Francis Hassard, Nathan Brown, John G Kenny, James E McDonald

**Affiliations:** 1 Forest Research, Centre for Forestry and Climate Change, Farnham, UK; 2 School of Biological Sciences, Bangor University, Bangor, UK; 3 School of Ocean Sciences, Bangor University, Bangor, UK; 4 Department of Computational and Systems Biology, Rothamsted Research, Harpenden, UK; 5 Centre for Genomic Research, Institute of Integrative Biology, University of Liverpool, Liverpool, UK

## Abstract

Decline-diseases are complex and becoming increasingly problematic to tree health globally. Acute Oak Decline (AOD) is characterized by necrotic stem lesions and galleries of the bark-boring beetle, *Agrilus biguttatus*, and represents a serious threat to oak. Although multiple novel bacterial species and *Agrilus* galleries are associated with AOD lesions, the causative agent(s) are unknown. The AOD pathosystem therefore provides an ideal model for a systems-based research approach to address our hypothesis that AOD lesions are caused by a polymicrobial complex. Here we show that three bacterial species, *Brenneria goodwinii*, *Gibbsiella quercinecans* and *Rahnella victoriana*, are consistently abundant in the lesion microbiome and possess virulence genes used by canonical phytopathogens that are expressed in AOD lesions. Individual and polyspecies inoculations on oak logs and trees demonstrated that *B. goodwinii* and *G. quercinecans* cause tissue necrosis and, in combination with *A. biguttatus*, produce the diagnostic symptoms of AOD. We have proved a polybacterial cause of AOD lesions, providing new insights into polymicrobial interactions and tree disease. This work presents a novel conceptual and methodological template for adapting Koch’s postulates to address the role of microbial communities in disease.

## Introduction

Trees are essential to landscape function and aesthetics, supporting diverse ecologies (Rackham, 2008) and providing key ecosystem services (Boyd *et al.*, 2013). However, significant areas of forest have been lost due to increasing outbreaks of disease and pest attack, and tree health is a current global concern (Cohen *et al.*, 2016). Tree diseases, including decline-diseases, are rising in profile due to an increased risk of introduction and spread through international plant trade and amplification effects of current and future climate change (McDowell *et al.*, 2011; Oliva *et al.*, 2014; Millar and Stephenson, 2015). Emerging evidence suggests that complex biotic interactions, including polymicrobial and insect activity, affect disease occurrence and severity (Adams *et al.*, 2013; Buonaurio *et al.*, 2015; Lamichhane and Venturi, 2015), yet little progress has been made in applying the latest advances in sequencing and culture-based methodologies to characterize pathosystems in trees. The prevailing paradigm of infection biology contends that one organism causes one disease (proved using Koch’s postulates). In contrast, there is increasing recognition of the importance of polymicrobial interactions in human disease, following developments in sequencing technologies that allow microbiome-wide association studies to identify the role of microbial communities in disease (Gilbert *et al.*, 2016). In the medical field, this is leading to adaptations of Koch’s postulates to include complex interactions between the environment, host and microbial communities (Hill, 1965; Falkow, 1988; Fredricks and Relman, 1996). However, progress in characterizing polyspecies interactions in plant disease has been limited, although there is clearly a need for contemporary approaches to investigating complex tree diseases (Lamichhane and Venturi, 2015). Analysis of such complex biotic interactions requires an integrated, systems approach, particularly in the case of decline-diseases where both complex abiotic and biotic interactions underpin disease development.

**Figure 1:**
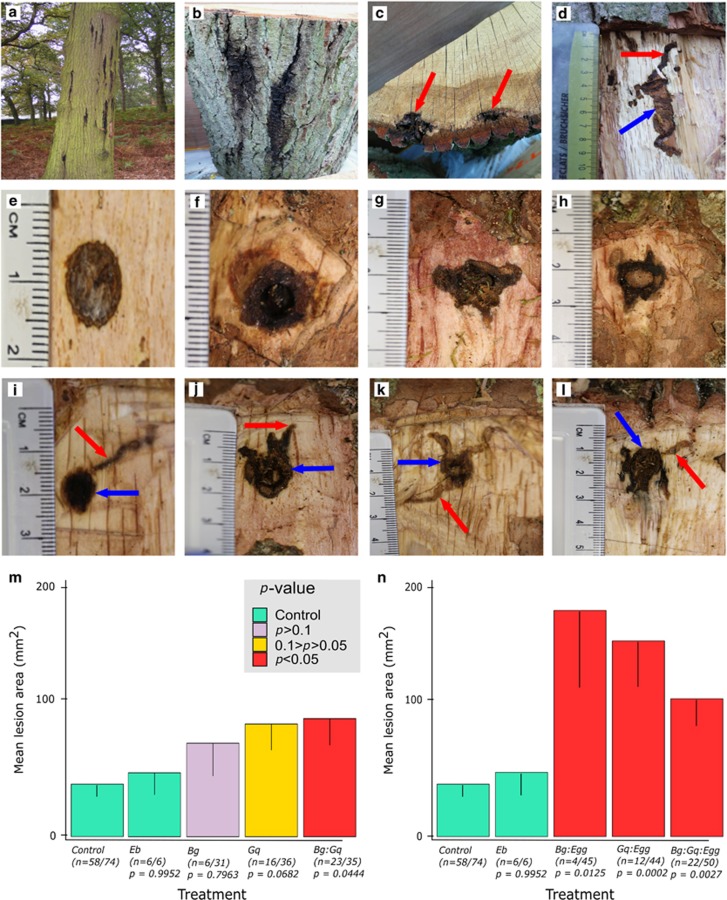
Symptoms of AOD observed in the field (**a**–**d**), reproducing the symptoms of AOD through log inoculations with combinations of bacteria and *Agrilus* larvae associated with AOD (**e**–**l**), and statistical analysis of lesion formation in log inoculations (**m**–**n**). (**a**) Stem bleeds symptomatic of AOD on a mature *Quercus robur* in the field. (**b**) Close up view of stem bleeds and bark cracks characteristic of AOD. (**c**) Cross-section through AOD stem bleeds, showing degradation of vascular tissue (red arrow). (**d**) Longitudinal section through an AOD stem bleed showing the association between bacterial lesions (blue arrow) and *Agrilus biguttatus* galleries (red arrow). (**e**) Wound response in the innerbark of the control treatment (inoculation of a wound with sterile water) of log inoculation trials. (**f**) Lesion formed in the innerbark of oak logs inoculated with *Brenneria goodwinii* in log inoculation trials. (**g**) Lesion formed in the innerbark of oak logs inoculated with *Gibbsiella quercinecans* in log inoculation trials. (**h**) Lesion formed in the innerbark of oak logs inoculated with a combination of *B. goodwinii* and *G. quercinecans* in log inoculation trials. (**i**) Wound response in the innerbark of the *A. biguttatus* treatment (inoculation of a wound with live eggs of *A. biguttatus*). Note the wound response (blue arrow) and clean gallery created by a larva (red arrow). (**j**) Lesion formed in the innerbark of oak logs inoculated with *B. goodwinii* and eggs of *A. biguttatus*. Note the lesion developing from the inoculation point (blue arrow) and from the galleries (red arrow). (**k**) Lesion formed in the innerbark of oak logs inoculated with *G. quercinecans* plus eggs of *A. biguttatus*. Note the lesion developing from the inoculation point (blue arrow) and from the galleries (red arrow). (**l**) Lesion formed in the innerbark of oak logs inoculated with *B. goodwinii*, *G. quercinecans* plus eggs of *A. biguttatus*. Note the lesion developing from the inoculation point (blue arrow) and from the galleries (red arrow). (**m**) Mean lesion area formed in the inner bark of logs inoculated with bacteria. Colour indicates statistical significance (see key). The bacterial species inoculated were back-isolated in fulfilment of Koch’s fourth postulate. Error bars=−s.e. (**n**) Mean lesion area formed in the inner bark of logs inoculated with bacteria and *A. biguttatus* eggs. Colour indicates statistical significance (see key in panel (**m**)). The bacterial species inoculated were back-isolated in fulfilment of Koch’s fourth postulate. *Eb*—*Erwinia billingiae*, *Bg*—*Brenneria goodwinii*, *Gq*—*Gibbsiella quercinecans*. Error bars=−s.e.

Decline-diseases, first formally described as a specific disease in the United States (Sinclair, 1965; Manion, 1981) but well documented elsewhere (Delatour, 1983; Sinclair and Lyon, 2005; Thomas, 2008) are of global concern (Pautasso *et al.*, 2015). Unlike most common tree diseases, decline-diseases are not caused by single primary pests or pathogens; instead they are complex syndromes, involving the sequential, combined and cumulative effects of (often secondary) biotic and abiotic agents (Manion, 1981; Thomas, 2008; Sallé *et al.*, 2014; Brown *et al.*, 2016). Currently, the United Kingdom is facing an episode of Acute Oak Decline (AOD), which occurs widely in southern and midland England, extending into Wales (Brown *et al.*, 2016; Denman *et al.*, 2016), and represents a significant threat to oak, particularly native species *Quercus robur* and *Quercus petraea* (Denman *et al.*, 2014). First recognized in Britain in the 1980s (Denman and Webber, 2009), similar declines have occurred in continental Europe (Hartmann *et al.*, 1989; Gibbs and Greig, 1997; Biosca *et al.*, 2003; Vansteenkiste *et al.*, 2004). AOD-affected trees show distinctive, weeping stem patches (stem bleeds), signifying areas of necrosis and fluid-filled cavities in the underlying inner bark (Denman *et al.*, 2014) (see Figures 1a–c), which disrupt vascular flow of nutrients and water essential to tree survival (Vansteenkiste *et al.*, 2004). Larval galleries of the buprestid beetle *Agrilus biguttatus* are found in conjunction with lesions (Brown *et al.*, 2015, 2017) (Figure 1d) and can also impact tree condition by girdling the tree when colonization is intense, leading to tree death (Sallé *et al.*, 2014). AOD typically affects mature oaks but has also been reported in young trees (Brown *et al.*, 2016).

First steps to determine the causes of stem bleeds led to the isolation of several novel bacterial species from AOD lesions, with three novel species, *Gibbsiella quercinecans* (*Enterobacteriaceae*) (Brady *et al.*, 2010), *Brenneria goodwinii* (*Pectobacteriaceae*) (Denman *et al.*, 2012; Adeolu *et al.*, 2016), *Rahnella victoriana* (*Yersiniaceae*) (Brady *et al.*, 2014; Adeolu *et al.*, 2016), and an unnamed *Pseudomonas* (Denman *et al.*, 2016; Sapp *et al.*, 2016) consistently isolated. Some of these species, for example, *G. quercinecans* as *Serratia* sp. (Biosca *et al.*, 2003; Poza-Carrión *et al.*, 2008) on *Quercus pyrenacia* and *Quercus ilex* are implicated as causative agents of stem bleeding on other oak species in Europe (Biosca *et al.*, 2003; Poza-Carrión *et al.*, 2008; Denman *et al.*, 2016). There was less consistent isolation of various other bacterial species, for example, *Lonsdalea quercina* ssp. *britannica* (Brady *et al.*, 2012), which is closely related to *Lonsdalea quercina* ssp. *quercina*, the causative agent of acorn gummosis on *Q. agrifolia* and *Q. wislizenii* in America (Hildebrand and Schroth, 1967), and several other novel species that are in the process of being formally described. Although correlation of certain bacterial species with AOD symptomology has been observed, empirical evidence on the causative agent(s) of AOD lesions is lacking and remains a barrier to developing informed management strategies for this disease. In the absence of a single putative primary pathogen as the causal agent of AOD lesions, we hypothesized that a polymicrobial complex is responsible for AOD lesion formation. However, demonstrating causation by a polymicrobial complex with associated insect activity on mature oak trees or logs is challenging in the context of fulfilling Koch’s postulates and requires a multi-faceted methodological and conceptual approach.

Here we applied a systems-level approach to determine cause(s) of necrosis in AOD using microbial isolation and culture, phenotypic tests, genomic analyses of *G. quercinecans*, *B. goodwinii* and *R. victoriana*, metagenome and metatranscriptome analysis of healthy and diseased trees and inoculation tests comprising both polybacterial inoculations and the addition of live *A. biguttatus* eggs to recreate the symptoms of AOD. This combined sequencing and cultivation-based approach provides a contemporary adaptation of Koch’s postulates to address the biotic components of a complex decline-disease and represents a conceptual model for future analyses of polymicrobial infections in trees and other systems.

## Methods

### Isolation of bacteria from healthy and diseased oak trees

In the search for putative causal agents of stem lesions, we wanted to determine the veracity of differences in occurrence and composition of bacterial communities in lesions and visually healthy trees, as the bacterial microbiome has previously been identified as the likely causal agent of stem lesions (Denman *et al.*, 2016). Conventional isolation and culture and microbiome analyses were used. Samples were acquired through citizen science reports (CSR), as well as structured studies (Denman *et al.*, 2016; Sapp *et al.*, 2016) ensuring the broadest possible coverage of AOD sites (Supplementary Table S1 and S2). Eighteen CSR sites were sampled (Supplementary Figure S1 and Supplementary Table S1), together with those from structured studies (Denman *et al.*, 2016; Sapp *et al.*, 2016), as well as five sites that had no history of AOD (N-AOD). Trees were sampled by forest pathologists who made site visits following the enquiry. In total, isolations from 66 trees were analysed. Destructive sampling of trees, by removing panels of diseased oak tissue, as well as healthy oak tissue from apparently healthy trees on the same sites, was carried out where permitted, and isolations were made using PYGA medium as described in Denman *et al.* (2014, 2016) and Sapp *et al.* (2016). Owing to the nature of the CSR studies, the number of tissue pieces plated out was variable, dependent upon the sample. Bacterial colonies emerging from chips of tissue were purified using standard streak-plating techniques; single visually representative colonies were selected, cultivated in Luria Bertani broth (LB) and identified with PCR and DNA amplicon sequencing as described in Denman *et al.* (2016).

### Statistical analysis of isolation study data sets

Bacterial yield between healthy and diseased trees was tested by fitting a generalized linear mixed-effects model with logit link function and binomial error distribution. Fixed effects were fitted for tree health and tissue position and random effects fitted for sites and trees within sites. Overdispersion in the model was taken into account by including an additional dispersion parameter.

Differences in bacterial communities were analysed by detrended correspondence analysis, down weighting bacterial species occurring in <5% of tissue combinations. Monte Carlo permutation tests were used to test for significant differences in bacterial communities between healthy and diseased trees and to test the effect of tissue position within the tree. Finally, Jaccard’s similarity index was used to identify any significant associations between bacteria across the 66 trees of the study.

### Genome sequencing of bacterial strains isolated from AOD-affected trees

#### Maintenance of bacterial strains used in genome analyses

*G. quercinecans* FRB97 (T) (Brady *et al.*, 2010), *B. goodwinii* FRB141 (T) (Denman *et al.*, 2012) and *R. victoriana* BRK18a (T) (Brady *et al.*, 2014) were isolated by Forest Research in the CSR studies from oak trees affected by AOD. Strains were stored in glycerol stocks at −80 °C and maintained on nutrient agar (Oxoid, ThermoFisher Scientific, Loughborough, UK) at 20 °C.

#### DNA preparation and genome sequencing on Pacific Biosciences RSII sequencing platform

The whole genomes of *G. quercinecans* FRB97, *B. goodwinii* FRB141 and *R. victoriana* BRK18a were sequenced using the Single Molecule Real-Time (SMRT) technology of the Pacific Biosciences RSII platform. A single colony of each isolate was sampled from nutrient agar (Oxoid) and used as inoculum for liquid culture, which was grown overnight in nutrient broth (Oxoid) at 28 °C, and shaken at 150 r.p.m. Total genomic DNA was isolated using the Gentra Puregene Yeast/Bact. Kit (Qiagen, Manchester, UK) and quantified using a Qubit fluorometer (Life Technologies, Paisley, UK). DNA integrity was assessed using 1% agarose gel electrophoresis. *G. quercinecans* and *B. goodwinii* DNA libraries were prepared using 10 μg of genomic DNA and sequenced by DUGSIM at Duke University, NC, USA, using P4/C2 chemistry and six SMRT cells per isolate. The *R. victoriana* DNA library was prepared and sequenced by the Centre for Genomic Research, University of Liverpool, UK using P6/C4 chemistry and one SMRT cell. All sequence data described in this study are available under BioProject PRJNA323828, (Supplementary Table S3). Genome assembly and annotation are described in Supplementary Methods.

### Metagenome sequencing of diseased and healthy oak trees

#### Collection of samples for metagenome sequencing

Tissue samples were collected from Runs Wood, Ross-on-Wye and two sites in Attingham park (Supplementary Table S2). More than half of the trees sampled for metagenome analysis were also sampled in the isolation study. A panel comprising all layers of stem tissue (outerbark, innerbark, sapwood and heartwood) was removed from the visible bleed area on each diseased tree or from stem areas at similar height on healthy trees according to previously described methods (Denman *et al.*, 2014, 2016; Sapp *et al.*, 2016). Samples were immediately flash frozen on dry ice and stored at −80 **°**C prior to processing.

#### Metagenome assembly, annotation and mapping to the genomes of *G. quercinecans* FRB97 (T), *B. goodwinii* FRB141 (T) and *R. victoriana* BRK18a (T)

Metagenomic reads were assembled using RAY-meta v2.3.1 (Boisvert *et al.*, 2012) using default parameters. Assemblies were annotated using Prokka v1.11 (Seemann, 2014). Translated annotations were aligned against the translated protein sequences of *G. quercinecans* FRB97 (T), *B. goodwinii* FRB141 (T), *R. victoriana* BRK18a (T) and two control genomes *Pectobacterium carotovorum* ssp. *carotovorum* PC1 and *Paenibacillus polymyxa* SC2 using BLASTx v2.2.26 (Altschul *et al.*, 1990). Metagenome sequences with >97% homology for at least 50 amino acids to proteins identified in *G. quercinecans*, *B. goodwinii* and *R. victoriana* were considered a match (Supplementary Table S4). Control genomes were selected to measure the stringency of alignment. *P. carotovorum* ssp. *carotovorum* is a canonical plant pathogen and a member of the soft-rot *Enterobacteriaceae* (SRE) (N.B. many members have recently been reclassified into novel families (Charkowski *et al.*, 2012; Adeolu *et al.*, 2016)), which had been identified sporadically and at low relative abundance from metagenomic taxonomic surveys within this study (Supplementary Table S5). However, *P. carotovorum* ssp. *carotovorum* had not previously been isolated from necrotic lesions on AOD-affected trees. Therefore, it was proposed that coding domain alignments to *P. carotovorum* ssp. *carotovorum* align to conserved genes, which are present in many *Enterobacteriaceae*, and their low relative abundance does not signify their presence but is an artefact of the alignment process. Resultant Circos plots agree with this proposal as conserved genes are frequently found in the metagenomic alignment against the *P. carotovorum* ssp. *carotovorum* PC1 genome, which are likely to align to other *Enterobacteriaceae*. Within *G. quercinecans*, *B. goodwinii* and *R. victoriana*, there is strong homology to conserved and variant genes. *P. polymyxa* SC2 was identified at low relative abundance in healthy and diseased metagenomic samples (Supplementary Table S5), therefore it was selected to test stringency of the metagenomic alignment and to measure its relative abundance in healthy and diseased microbiomes using an alternative method. Homologous bacterial protein identities and the workflow used for metagenomic analysis is available from GitHub: (https://github.com/clydeandforth/multi_omics_study.git).

#### Taxonomic classification of metagenome sequences

**Figure 2:**
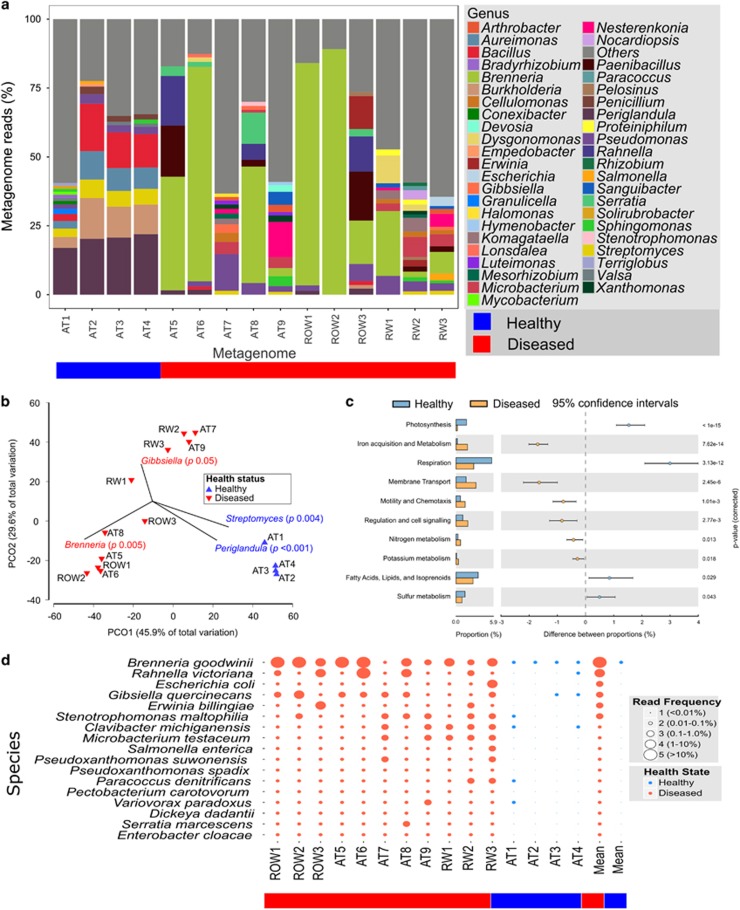
Comparative metagenomic analysis of the taxonomic composition and function of the oak microbiome in healthy tissue (from trees without AOD) and diseased tissue (from trees with AOD). (**a**) The plot depicts the genera represented in all metagenomes based on One Codex binning of raw reads, demonstrating a clear shift in the microbial community between healthy and diseased trees. (**b**) *Brenneria* and *Gibbsiella* are statistically correlated to diseased tissue, as shown by a principal coordinate ordination analysis based on statistics calculated by Primer v7 and PERMANOVA+ using One Codex binning data. The first two axes depict the plotted community composition. Correlation vectors in the graph are significant using an *R*^2^>0.05. A Welch’s *t*-test was performed to test significance of differences between key taxa (identified above) and between healthy and diseased trees (pooled abundances for each factor). Resultant *P-*values from Welch’s *t*-test are overlaid on the correlation biplot. (**c**) Gene groups involved in bacterial phytopathogenic activity are significantly increased in AOD diseased trees, as shown by comparative functional analysis of SEED subsystem categories as annotated by MG-RAST on assembled metagenome contigs. The analysis of SEED subsystem metagenome data was performed using Stamp. Statistically significant functional differences between diseased and healthy communities were calculated using *G*-test with Yates’ correction. The Newcombe–Wilson test was performed to calculate confidence intervals between two binomial population proportions. (**d**) The genomes of 17 species were found to be common across all AOD metagenomes. Visualization of Kraken metagenome analysis of stem samples demonstrates the shifts in bacterial microbiome compositions. Bubble sizes are categorized based on the relative percentage frequencies of Kraken species-level alignment of raw metagenome sequencing reads and are depicted in the figure as 1 (read frequency <0.01), 2 (read frequency 0.01–0.1), 3 (read frequency 0.1–1), 4 (read frequency 1–10) and 5 (read frequency >10). Red bubbles signify samples from diseased trees, while blue bubbles signify samples from healthy trees. The 17 common species were determined based on common occurrence across all diseased tissue samples. Additionally, six species more abundant among the healthy trees were included to provide a contrasting shift.

To compare the taxonomic composition of the oak microbiome, raw sequence reads were taxonomically labelled using Kraken v0.10.5 beta (Wood and Salzberg, 2014) and One Codex (Minot *et al.*, 2015) (Figure 2a). Taxonomic labelling using Kraken was performed on the standard RefSeq genome database supported by Kraken, with the addition of the genomes of *B. goodwinii* FRB141 (T), *R. victoriana* BRK18a (T), *G. quercinecans* FRB97 (T) and *Lonsdalea quercina* ssp. *quercina* ATCC 29281 (Figure 2d and Supplementary Table S5). Taxonomic analysis using One Codex was conducted against the One Codex March ’16 Preview database with the addition of the genomes of *G. quercinecans*, *B. goodwinii* and *R. victoriana*.

#### Functional annotation of metagenome sequences

Metagenome datasets derived from samples AT2, AT3, AT4, AT5, AT6, ROW1, ROW2 and ROW3 were analysed via MG-RAST (Meyer *et al.*, 2008) using Hierarchical Classification against the Subsystems database with an *E*-value cutoff of 1e-5, a minimum percentage identity cutoff of 80% and a minimum alignment length of 50. Descriptions of the taxonomic and functional composition of the metagenomes derived from MG-RAST were comparable with those derived from analysis of the same data sets using One Codex and Kraken and further validated by mapping of metagenome reads against the finished genomes of *B. goodwinii, G. quercinecans* and *R. victoriana*.

#### Statistical analysis of metagenome data sets

Statistical analyses were performed using Primer v7 (Clarke and Gorley, 2015) with PERMANOVA+ add on to explore relationships between community changes (Figure 2b). One Codex metagenome data was log (*N*+1) transformed to downweight the most abundant genera. Next, dissimilarities were calculated with the S17 Bray–Curtis similarity coefficient. A principal coordinate ordination analysis was performed by plotting the interpoint dissimilarity values for each factor (site and disease status), and the variation in community composition was plotted as the first two axes (preserving actual dissimilarities) (Gower, 1966). A correlation was performed between each taxon and each community coordinate. Correlations with each component were deemed significant (*R*^2^>0.5) and a vector biplot was overlaid to visualize the strength of the correlation. A Welch’s *t*-test was performed to test significance of differences between key taxa (identified above) and between healthy and diseased trees (pooled abundances for each factor). Resultant *P*-values from Welch’s *t*-test are overlaid on correlation biplot with significance at >95% (*P*<0.05) deemed significant. Comparative functional analysis of MG-RAST (Meyer *et al.*, 2008) annotated, metagenome data was performed using Stamp v2.1.3 (Parks *et al.*, 2014). Statistically significant functional differences between diseased and healthy communities were calculated using *G*-test with Yates’ correction. The Newcombe–Wilson test was performed to calculate confidence intervals between two binomial population proportions (Brown and Li, 2005).

### Metatranscriptome sequencing of AOD diseased oak trees

#### Collection of samples for metatranscriptome sequencing and RNA extraction

For RNA sampling, two separate lesions (samples AT11 and AT12) from a single tree were analysed in June 2013. Swabs of the lesion fluid were collected in addition to tissue from the active margins of the lesion and immediately frozen in liquid nitrogen and transported back to the laboratory in a vessel containing liquid nitrogen. Samples were stored at −80 **°**C prior to processing. Before RNA extraction, samples in liquid nitrogen were ground with a pestle in a mortar to homogenize the tissue. RNA was extracted from 2 g of tissue using the PowerSoil Total RNA Isolation Kit (MoBio, Cambridge, UK) according to the manufacturer’s instructions. RNA was quantified using a Qubit fluorometer (Thermo Fisher, Warrington, UK) and quality assessed using the Bioanalyser 2100 (Agilent, Manchester, UK).

#### Metatranscriptome sequencing

Sequencing libraries were prepared from samples of total RNA using the strand-specific ScriptSeq Preparation Kit (Illumina, Cambridge, UK) and sequenced using 2 × 100 bp paired-end sequencing on the Illumina HiSeq platform. Reads were trimmed using first Cutadapt 1.2.1 (Martin, 2011) and additionally Sickle 1.2.00 (Joshi and Fass, 2011). Owing to low RNA yields from the lesion samples, total RNA was sequenced and rRNA sequence reads were subsequently depleted *in silico* prior to mRNA transcript analysis.

#### Metatranscriptome assembly and functional annotation

Two *in silico* rRNA-depleted metatranscriptome libraries were aligned to the *G. quercinecans* FRB97 (T), *B. goodwinii* FRB141 (T), *R. victoriana* BRK18a (T) and control genomes (*P. carotovorum* ssp. *carotovorum* PC1 and *P. polymyxa* SC2) (Supplementary Figures S2 and S3) with Bowtie2 v2.2.4 (Langmead and Salzberg, 2012), using local mode to maximize alignment score. Aligned reads were converted from Sequence Alignment/Map (SAM) to Binary Sequence Alignment/Map (BAM) format and indexed using SAMtools v1.2 (Li *et al.*, 2009). To avoid false positives in the detection of gene expression, a gene was considered as being expressed if ⩾3 transcripts were aligned and the combined coverage from both libraries represented >20% of the gene, (adapted from (Versluis *et al.*, 2015) (Figures 2a–c and Supplementary Figure S4). A custom Perl script was designed to extract transcript alignments and is available from GitHub: (https://github.com/clydeandforth/multi_omics_study.git). Aligned transcripts were visualized in Artemis (Carver *et al.*, 2012).

#### Log inoculations

Pathogenicity tests were set up to reproduce lesions characteristic of AOD under controlled conditions. The following hypotheses were tested: (1) Key species consistently isolated from AOD symptomatic oak can cause necrosis of oak stem tissue, (2) Combinations of key bacterial species cause more severe tissue necrosis (reflected in larger lesions), than individual species alone, and (3) The interaction between *A. biguttatus* larvae (derived from eggs) and bacteria leads to the development of AOD symptoms.

Four experiments were carried out over 3 consecutive years as testing could only be carried out annually when beetle eggs were available. Three trials were carried out on oak logs in growth chambers, and the fourth trial was set out in the field, where stems of young plantation-oak (25 years old), were used instead of logs.

#### Growth chamber log trials

Logs used in the trials were obtained from freshly felled *Q. robur* trees, with diameter at 1.3 m (DBH)=16–20 cm. The trees were located in the Straits Enclosure of the Alice Holt Forest, Hampshire, England, UK, and logs were transported to the laboratory after felling where they were prepared for inoculation. Logs measuring 40 cm (mini-logs) or 130 cm (long logs) in length had the uppermost cut surface sealed with isoflex liquid rubber (Ronseal Ltd, Chapeltown, Sheffield) to prevent desiccation. Logs were placed, lower cut surface down, in saucers containing water. Logs were inoculated in August or September and incubated at 25 °C in the growth chamber with a 12 h photoperiod. Mini-logs were used for all treatments involving *A. biguttatus* eggs; in each case, only one bacterial+eggs treatment type per mini-log was tested to guard against cross-contamination through larval spread (Supplementary Table S2). All the bacterial treatments without eggs (either single species or combinations of species) were placed on the same long log, with inoculation points marked out along its length. Eggs of *A. biguttatus* were produced in Forest Research’s laboratories at Alice Holt (Reed, 2016). In a single experiment (2014 NW; Supplementary Table S2), non-wound inoculations were carried out; the remaining two growth chamber trials and the field trial were inoculated using shallow wounds to the outer surface of the innerbark, made by a 10 mm cork-borer. Half a loop of inoculum scooped from 24-h-old inoculum plates using disposable plastic loops was inserted into the wound and rubbed to dislodge the bacterial cells around the wound surface. The outer bark plug was replaced on the inoculation point, wounds covered with parafilm and damp cotton wool and sealed with duct tape. After 4 months of incubation, experiments were terminated, outer bark stripped from inoculation points to expose lesions, which were hand traced onto tracing paper, and back isolations were cultured onto peptone yeast glucose agar. Mass bacterial colonies that developed were tested for the presence of *B. goodwinii*, *G. quercinecans*, *Rahnella* and *Lonsdalea* using a multiplex Taqman qPCR assay (Roche, Brighton, UK). Lesion areas were calculated using ASSESS V2 (APS, Minneapolis, MN, USA).

### Statistical analyses

#### Log lesion areas

The four pathogenicity trials were used to establish the impact of different bacterial species tested individually, in combination (polybacterial inoculations) and with or without the addition of *A. biguttatus* eggs (Supplementary Table S2) on tissue necrosis (assessed by the size of the lesion area associated with each inoculation point).

Lesion area data were refined in a hierarchical manner, such that the following data sets were used for lesion area analysis:

1. Non-contaminated samples fulfilling Koch’s postulates, with galleries present (for *A. biguttatus* samples indicating that eggs had hatched);
2. Non-contaminated samples fulfilling Koch’s postulates, with or without galleries present (for *A. biguttatus* samples); and
3. Non-contaminated samples.

Further information on statistical analyses of log lesion areas are described in Supplementary Methods.

## Results

### Isolation study

In the isolation study, analysis of 38 diseased trees from 23 sites, plus 13 healthy trees in 11 of these sites, and 15 healthy trees from five sites with no history of AOD identified 159 bacterial taxa. Higher yields of bacteria were obtained from lesion margin tissue of symptomatic trees compared with healthy tree tissue (F_1,28_, *P*<0.001), and the lesion margin bacterial profile was significantly different to healthy tissues (Monte-Carlo permutation test of 1000 permutations, *P*<0.001) (Supplementary Figure S5 and Supplementary Table S6). Key genera isolated included *Pseudomonas* (comprising multiple taxa and occurring in healthy as well as diseased trees); but *Gibbsiella*, *Brenneria* and *Rahnella* dominated lesion margins (Supplementary Table S6). *G. quercinecans* occurred on all disease sites and was isolated from 83% of diseased and 4% of healthy trees sampled, comprising 17% of total diseased samples and <0.1% of total healthy samples. *B. goodwinii* was more difficult to isolate but was obtained from 15 sites, accounting for 16% of total diseased and <0.1% of total healthy samples. Members of the genus *Rahnella* were obtained from 65% of diseased sites, but *R. victoriana* was isolated only from diseased trees (37%), on nine of the sites. *L. quercina* ssp. *britannica* (Brady *et al.*, 2012) was isolated sporadically on four sites, and a *Pseudomonas* species (*P. fulva*-like) not yet formally identified, was isolated at eight sites from diseased trees only (Supplementary Table S6). There was a significant co-occurrence of *G. quercinecans* and *B. goodwinii* in diseased tissue (*J*=0.56, *P*<0.001), but neither was isolated from trees on sites with no history of AOD.

### Oak microbiome analysis

Taxonomic analysis of the metagenome using unassembled metagenome sequence reads against the One Codex (Minot *et al.*, 2015) March ’16 Preview database (with the addition of the genomes of *G. quercinecans*, *B. goodwinii* and *R. victoriana*) also revealed a shift in microbiome composition between healthy stem tissue and AOD lesions (Figures 2a and b). *Periglandula*, *Burkholderia*, *Streptomyces*, *Bacillus* and *Auriemonas* were the most abundant genera in healthy trees, whereas *Brenneria* dominated diseased tissue (mean read abundance, 37.5%) (Figure 2a). The mean read abundance of *Gibbsiella* (0.9%) and *Rahnella* (3.7%) was also greater in diseased tissue when compared with healthy tissue (both 0.1%). *Pseudomonas*, a diverse genus comprising both endophytic and phytopathogenic bacteria (Vinatzer *et al.*, 2014), had similar mean abundances in both diseased (4.8%) and healthy trees (3.3%). Correlation coefficients and Welch’s unequal variances *t*-tests revealed that *Streptomyces* (*t*_Welch’s_*=*49.7, *P*=0.004) and *Periglandula* (*t*_Welch’s_*=*821.8, *P*<0.001) were significantly associated with healthy trees, whereas *Brenneria* (*t*_Welch’s_*=*12.4*, P*=0.006) and *Gibbsiella* (*t*_Welch’s_*=*4.7, *P*=0.05) were strongly correlated with the lesions of diseased trees (Figure 2b and Supplementary Table S7). *Pseudomonas* and *Rahnella* were not strongly correlated with either health state (Supplementary Table S7).

To identify the most abundant species in the lesion microbiome, raw metagenome reads were aligned using Kraken (Wood and Salzberg, 2014) against reference genome databases (see Methods section), revealing 17 genomes that were commonly detected in diseased tissue (Figure 2d and Supplementary Table S7). *B. goodwinii*, *G. quercinecans* and *R. victoriana* were detected in the lesion metagenome of all trees with AOD. Overall, *B. goodwinii* was the most abundant genome in the lesion microbiome (range, 0.3–49% of metagenome sequence reads; mean, 12%) but was also detected in much lower proportions in healthy trees (0.01% in all samples). *R. victoriana* (range, 0.01–15% mean, 2.1%) and *G. quercinecans* (range, 0.02–0.8% mean, 0.3%) were the second and fourth most abundant genomes in the lesions of diseased trees, respectively. Functional metagenome analysis (Figure 2c and Supplementary Figure S6) revealed that genes associated with carbohydrate metabolism, membrane transport and virulence, defence and disease are key features of the lesion microbiome, suggesting that many of these functions are encoded in the genome of *B. goodwinii*, *G. quercinecans* and *R. victoriana*.

### Genome analysis of AOD-associated bacteria

**Figure 3:**
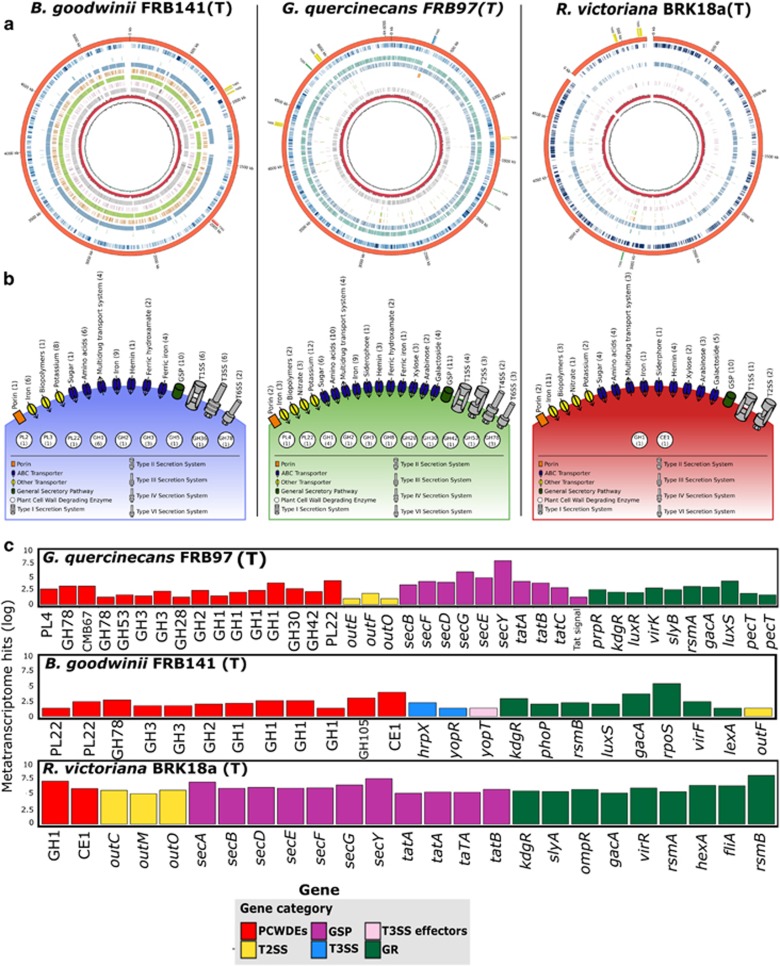
Functional genome analysis of *Gibbsiella quercinecans* FRB97 (T), *Brenneria goodwinii* FRB141 (T) and *Rahnella victoriana* BRK18a. (**a**) Circular representations of *B. goodwinii* FRB141, *G. quercinecans* FRB97 and *R. victoriana* BRK18a genomes. From outside to inside, circles represent: (1) Assembled bacterial genomes, outermost (orange) circle, with encoded secretion system annotated at their genomic loci. (2) Metatranscriptome heatmap. Alignment of two *in silico* combined metatranscriptomes recovered from two necrotic lesions of AOD-affected trees against the bacterial genomes. Blue saturation represents increasing transcript alignments. (3–9) Seven metagenomes from necrotic lesions on AOD-affected trees and one healthy tree (metagenomes were extracted from two sites, Attingham and Runs Wood) were aligned through their coding domains to homologous regions in the bacterial genomes. (3) Attingham healthy (AT1) aligned metagenome-coding domains (light purple). (4) Attingham diseased (AT7) aligned metagenome-coding domains (aqua). (5) Attingham diseased (AT8) aligned metagenome-coding domains (blue). (6) Attingham diseased (AT9) aligned metagenome-coding domains (orange). (7) Runs Wood diseased (RW1) aligned metagenome-coding domains (green). (8) Runs Wood diseased (RW2) aligned metagenome-coding domains (pink). (9) Runs Wood diseased (RW3) aligned metagenome-coding domains (grey). (10) G+C content across the bacterial genome. (11) G+C skew across the bacterial genome. (**b**) Schematic diagram of transporters and transported proteins recovered from lesion metatranscriptomes and aligned against each bacterial genome. The number of significantly expressed (>3 aligned transcripts, covering >20% of the gene) genes is shown in parentheses. (**c**) Number of transcripts aligned against selected virulence genes encoded within *B. goodwinii* FRB141, *G. quercinecans* FRB97 and *R. victoriana* BRK18a. Gene categories are represented by the following colours: red—PCWDE, purple—general secretory pathway (GSP), yellow—type II secretion system (T2SS), blue—type III secretion system (T3SS), pink—type III secretion system effectors (T3SS effectors), and green—global regulators (GR).

Whole-genome sequencing of *B. goodwinii* FRB141, *G. quercinecans* FRB97 and *R. victoriana* BRK18a (Supplementary Table S8) revealed that they are phylogenetically related to opportunistic phytopathogens belonging to the SRE (Charkowski *et al.*, 2012; Adeolu *et al.*, 2016) and possess a catalogue of virulence genes associated with canonical phytopathogens (Supplementary Table S9). The SRE alternate their lifestyle from benign commensals to brute force necrotrophic pathogens, which macerate cell wall polysaccharides by releasing plant cell wall-degrading enzymes (PCWDEs) (Toth *et al.*, 2006). A genome-wide search for PCWDEs in *G. quercinecans*, *B. goodwinii* and *R. victoriana* revealed the presence of pectinases, cellulases and tannases (Figure 3b, Supplementary Table S9) whose activity has been confirmed phenotypically (Supplementary Figure S7). Furthermore, *G. quercinecans* and *R. victoriana* possess a type II secretion system, an operon which releases most PCWDEs and is therefore the central virulence facilitator of necrotrophic plant pathogens. *B. goodwinii* encodes a type III secretion system, the principal virulence factor of established hemibiotrophic pathogens such as *Pseudomonas syringae*, which use the operon to evade immune surveillance, allowing bacteria to increase their numbers before releasing necrotic enzymes as nutrients are depleted (Tampakaki *et al.*, 2010).

To address the role of *B. goodwinii*, *G. quercinecans* and *R. victoriana* in the aetiology of AOD, we aligned metagenome sequences and transcripts recovered from necrotic lesions of AOD-affected trees against their genomes (Figure 3a). Alignment of six AOD lesion metagenomes revealed an average of 2225 homologous proteins in *B. goodwinii* FRB141, 858 in *G. quercinecans* FRB97 and 396 in *R. victoriana* BRK18a (Supplementary Table S4). Furthermore, annotated genes from the assembled metagenome of a healthy oak revealed only two homologous proteins in *B. goodwinii* FRB141, one in *G. quercinecans* FRB97 and two in *R. victoriana* BRK18a. Lesion transcripts were aligned to coding regions within *B. goodwinii* FRB141, *G. quercinecans* FRB97 and *R. victoriana* BRK18a; this revealed that the transcripts aligned significantly to virulence genes, including PCWDEs, secretion system machinery and regulators of PCWDEs (Figure 3c and Supplementary Figure S4). *G. quercinecans* FRB97 expressed the pectic enzymes, polygalacturonase and rhamnogalacturonate lyase, and β-glucosidase (cellulase), pectate exo-lyase and oligogalacturonide lyase (which cleaves polygalacturonic acid, the by-product of pectin lyase) (Moran *et al.*, 1968). *B. goodwinii* FRB141 expressed phosphocellosbiose, β-galactosidase and several type III secretion system effectors, *R. victoriana* BRK18a expressed many general secretory pathway genes, a β-glucosidase, a tannase and carbohydrate esterase enzyme (Yao *et al.*, 2013). Key regulators of pectinolysis were expressed in all three bacteria, including *kdgR*, *phoP*, *pecT*, *rsmA*/*rsmB* and *gacA* (Figure 3a).

### Log lesion areas

Figures 1e–l show lesions caused by bacteria with or without *A. biguttatus* inoculated into logs in pathogenicity tests. Notable results for lesion area analysis are presented in Figures 1m and n (non-contaminated samples fulfilling Koch’s postulates, with galleries present for *A. biguttatus* samples). In bacteria-only inoculations, lesions significantly bigger than wound controls (Figure 1m) were made by the combination of bacterial species *B. goodwinii*+*G. quercinecans* (*t*_dunnetx_=2.97, *P*=0.044); the bacterial species *G. quercinecans* had larger lesion areas versus the controls, although this was not significant and the *P*<0.05 level (*t*_dunnetx_=2.79, *P*=0.068); *B. goodwinii* lesions were smaller and not significantly different from the controls in terms of mean lesion area (*t*_dunnetx_=1.30, *P*=0.796), but the necrosis was clearly different to the control wound response (see Figure 1e vs f). Inoculation with *Erwinia billingiae*, a known ubiquitous non-pathogenic bacterium, served as a negative control demonstrating lesion area no different to the control wound response. The pattern strengthened when bacteria plus *A. biguttatus* (applied as eggs) were co-inoculated (Figure 1n). Lesions created by *B. goodwinii* and *A. biguttatus* eggs (larvae) were significantly bigger than wound controls (*t*_dunnetx_=3.40, *P*=0.0125), as were *G. quercinecans* and *A. biguttatus* (*t*_dunnetx_=4.65, *P*=0.0002) and the combination of *B. goodwinii*+*G. quercinecans*+*A. biguttatus* (*t*_dunnetx_=3.92, *P*=0.0027) (Figure 1n). Bacteria were re-isolated from lesion margins and at intervals along the larval galleries, which were becoming necrotic-forming part of the lesion. Apart from demonstrating the necrogenic ability of the bacteria, these results showed that the larvae and larval galleries have an important part in increasing lesion area, implicating them in the spread of bacteria within infected trees.

### Bacterial-positive back-isolation and A. biguttatus contamination

There was a high success of back-isolation for both *B. goodwinii* and *G. quercinecans* when these species were included as treatments (70% and 80%, respectively); both species were also identified as contaminants (that is, back-isolated when not part of the treatment inoculation; *B. goodwinii*=9% *G. quercinecans*=16%), although the contamination rate was significantly lower than treatment back-isolation in both cases (*B. goodwinii: z*=7.98, *P*<0.001; *G. quercinecans: z*=6.65, *P*<0.001). For *G. quercinecans*, the rate of contamination was affected by the presence of *A. biguttatus* eggs: when eggs were absent *G. quercinecans* contamination was 3% when eggs were present this rose to 38% contamination, a significant increase (*z*=3.59, *P*<0.001). There was no significant effect of *A. biguttatus* eggs on *B. goodwinii* contamination (eggs absent, *B. goodwinii* contamination=2% eggs present, *B. goodwinii* contamination=7%, *z*=1.60, *P*=0.11).

## Discussion

Fulfilling Koch’s postulates represents the traditional paradigm for proving disease causation. However, contemporary molecular approaches have transformed our appreciation of the role of microbial communities and polymicrobial interactions in disease. There are situations where the one pathogen causes one disease model is not suitable to prove causality and must be adapted to accommodate polymicrobial infections. For example, in decline-diseases of trees, application of Koch’s postulates in the strictest sense cannot fully address proving causality of lesion formation as more than one necrogenic agent is involved in the disease syndrome. Here causation of AOD lesions was demonstrated using a combined cultivation-based and sequencing approach in a dynamic adaptation of Koch’s postulates analogous to that described by Byrd and Segre (2016). Koch’s first postulate is fulfilled by the consistent isolation and metagenomic detection of *G. quercinecans*, *B. goodwinii* and *R. victoriana* in trees affected by AOD.

This combined approach delivered an improved understanding of lesion microbiome composition, as some species were difficult to isolate and culture. For example, *B. goodwinii* and *Lonsdalea quercina* ssp. *britannica* were under-represented, whereas *G. quercinecans*, which is more amenable to isolation and cultivation, was overrepresented, with the opposite trend evident in metagenomic studies. Furthermore, *G. quercinecans*, *B. goodwinii* and *R. victoriana* were absent on sites with no history of AOD, were rarely isolated from healthy oak on diseased sites and had negligible abundance in healthy metagenome samples at AOD sites, complying with Koch’s second postulate. Ultra-low levels of *G. quercinecans*, *B. goodwinii* and *R. victoriana* were detected in some of the healthy trees but only from sites where AOD is present, raising interesting questions about their existence in the wider environment. Possible explanations for the presence of *G. quercinecans*, *B. goodwinii* and *R. victoriana* in healthy trees on AOD sites may include; (1) the tree is in early stages of lesion formation, where visible symptoms have not yet developed, or that (2) *G. quercinecans*, *B. goodwinii* and *R. victoriana* are endophytes or epiphytes that opportunistically multiply after tissue necrosis is initiated by another organism. In the first situation, the chances of detecting asymptomatic lesion formation seem fairly remote, especially as crown condition is not a reliable indicator of tree predisposition status in the early stages of decline development. In the second case, several pieces of evidence counteract the possibility that these organisms multiply opportunistically after tissue necrosis occurs, as (1) we observed the expression of putative necrogenic enzymes and virulence factors of these bacteria in AOD lesions *in planta*, implicating them in having an active role in tissue degradation, and (2) *G. quercinecans* and *B. goodwinii* caused significant lesion formation in log inoculation trials, demonstrating actual lesion-forming capabilities. It is important to note that the oak logs and trees used in our trials were selected from areas with no history of the disease.

Furthermore, all three bacterial species possess the genomic capability to cause tissue necrosis, as determined through genomics, *in situ* functional metagenomics and metatranscriptomics. Inoculation onto live oak logs confirmed significant necrogenic ability of *B. goodwinii* and *G. quercinecans*, but further work with *R. victoriana* inoculation is required. Thus the pathogenic phenotype of *B. goodwinii* and *G. quercinecans* has been confirmed, broadly fulfilling Koch’s third postulate that inoculation tests can cause the disease anew. However, bacterial species combinations caused even greater necrosis indicating cumulative effect and possible synergism, this is a contemporary adaptation of Koch’s postulates *sensu stricto*. Finally, once inoculated onto live oak panels, *B. goodwinii* and *G. quercinecans* were re-isolated to fulfil Koch’s fourth postulate (although back-isolation of some species was difficult, and there is a need for developing rapid, cost-effective tools to detect pathogens that are difficult to isolate). Consequently, we propose that the biotic component of the AOD lesions is a polybacterial complex comprised primarily of *G. quercinecans* and *B. goodwinii,* which are now established as key causal agents of tissue necrosis, the primary symptom of AOD. Our studies also indicated that other members of the microbiome may contribute to the pathology of AOD. Microbiome analysis suggested that *R. victoriana* is abundant and also important in AOD lesions; however, back-isolation of *R. victoriana* was variable, and further tests to characterize its role and interactions with *B. goodwinii* and *G. quercinecans* are required. In addition, *Lonsdalea quercina* ssp. *britannica* in particular demonstrated variable but at times virulent pathogenicity in log inoculations, although it was not consistently present in AOD lesions. Our results indicate that *Agrilus* larvae potentiate the spread of these necrogenic bacterial species within tree tissue, generating new break-out points of tissue necrosis and replicating the observed aetiology of AOD. Ultimately, vascular degradation arising from a combination of bacterial tissue necrosis and inner bark damage from larval galleries exacerbates and accelerates the decline by interrupting carbon resource allocation, preventing accumulation in roots and reducing water availability.

It is clear that, where possible, microbiome analysis methods together with inoculation assays should become the accepted standard for profiling disease complexes, particularly when considering complex interactions between microorganisms, insects, the environment and the host. This phenomenon has never previously been addressed in arboreal systems using the approaches described here. Our work therefore highlights the importance of a systems-level approach for characterizing the pathology of complex diseases and represents a conceptual and methodological template for adapting Koch’s postulates to address the role of microbial communities in disease. In recent years, microbiome studies have transformed our understanding of the role of human gut microbiota in a variety of conditions, including bowel and cardiovascular disease (Frank *et al.*, 2007; Koeth *et al.*, 2013), and consequently, microbiome-wide association studies that link microbial consortia to disease will have an important role in the development of future diagnostics and therapies for disease (Gilbert *et al.*, 2016). For decline-diseases in particular, further adaptation may be required to include the role of host predisposition.

Although AOD has been formally described as a major threat to UK oak, similar decline-diseases have been reported in mainland Europe (Delatour, 1983; Hartmann *et al.*, 1989; Biosca *et al.*, 2003; Vansteenkiste *et al.*, 2004; Poza-Carrión *et al.*, 2008), the Middle East and the Americas (Lynch *et al.*, 2014), indicating that AOD is a global concern that has likely evaded attention due to the complexity of its causative agents. The polybacterial nature of AOD exhibits similarities to other economically important tree diseases such as olive knot disease (Buonaurio *et al.*, 2015) but may also be applied to other complex diseases. Generally, our findings highlight the importance of understanding polymicrobial interactions in the context of future-proofing plant health to protect important but increasingly disease-prone forests and crops that are a fundamental part of our global landscape.
