# Supplementary figures and images for: Microbiome and infectivity studies reveal complex polyspecies tree disease in Acute Oak Decline
Source: ISME J. 2017 Oct 13;12(2):386–99. doi: 10.1038/ismej.2017.170 (PMC5776452; doi:10.1038/ismej.2017.170)

**
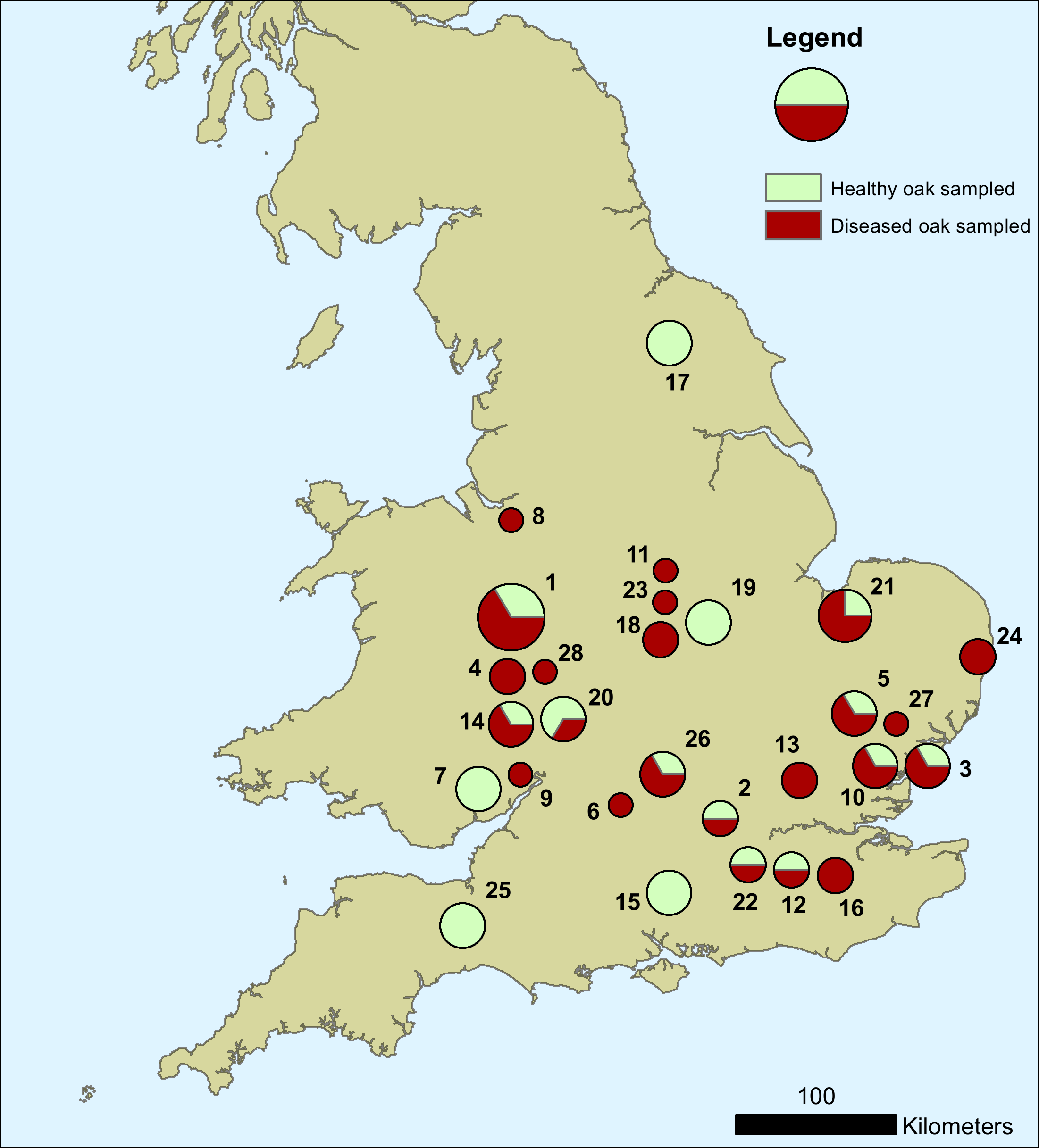
**

Supplement: Supplementary Figure S1 [file ismej2017170x2.docx]

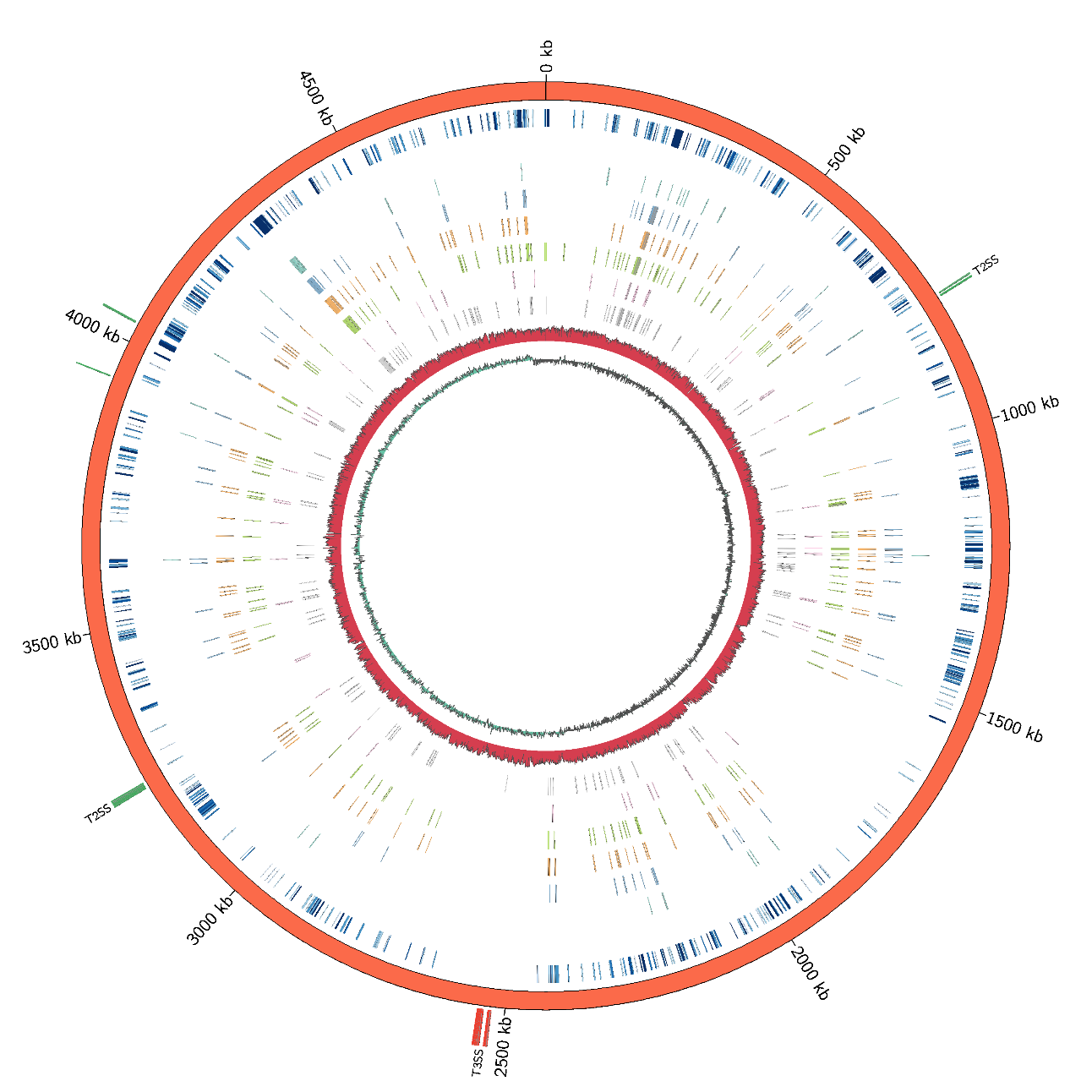

Supplement: Supplementary Figure S2 [file ismej2017170x3.docx]

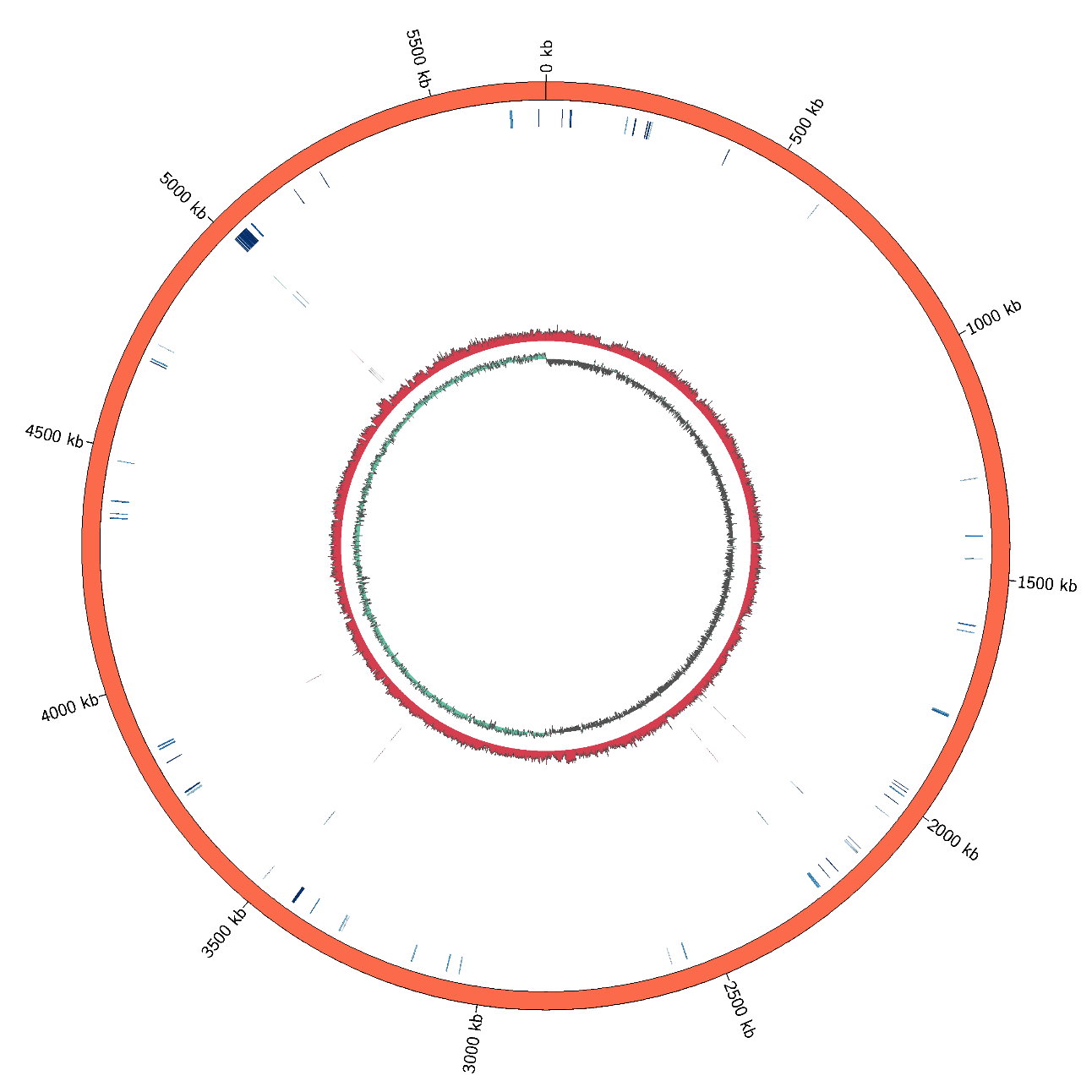

Supplement: Supplementary Figure S3 [file ismej2017170x4.docx]

**
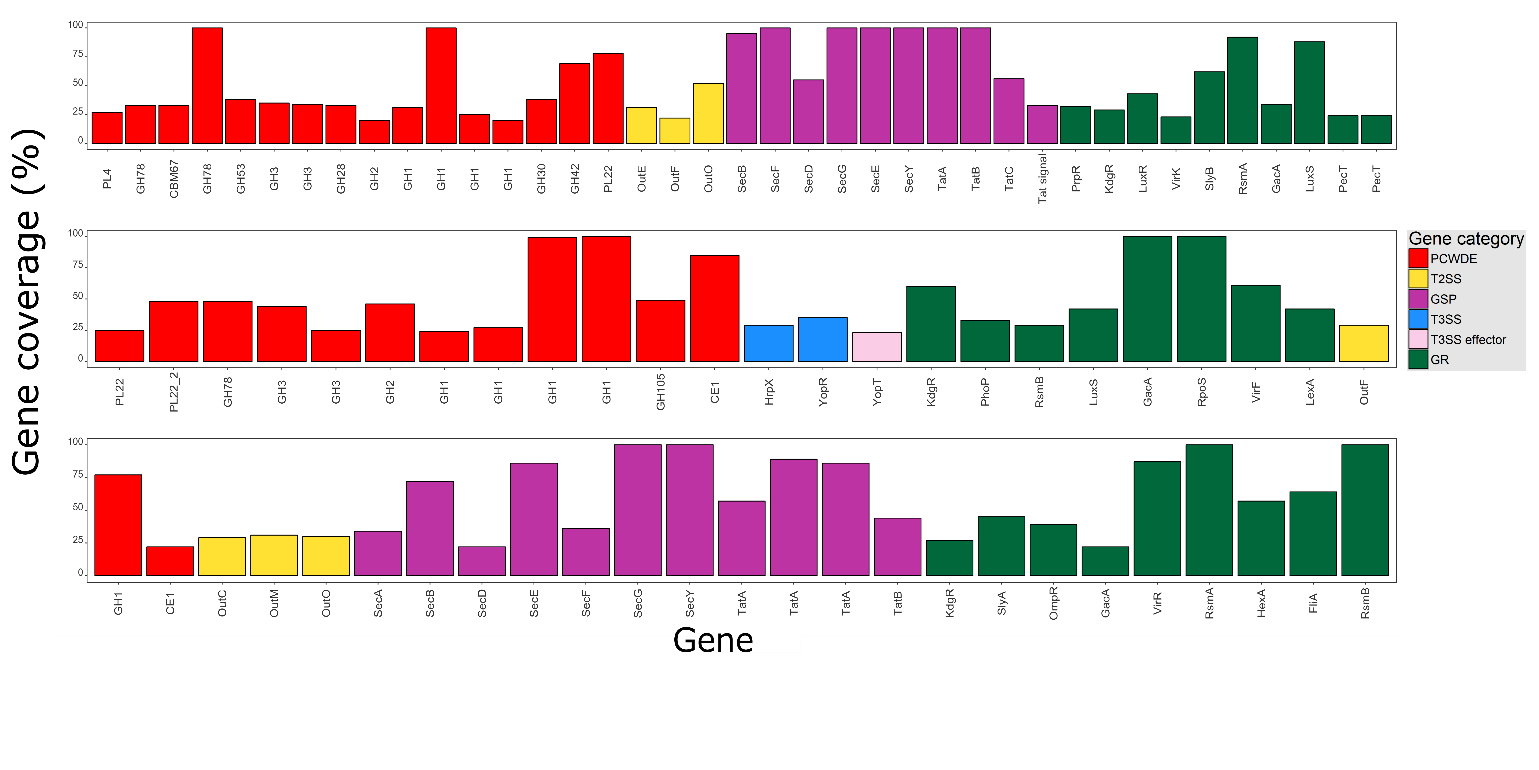
**

Supplement: Supplementary Figure S4 [file ismej2017170x5.docx]

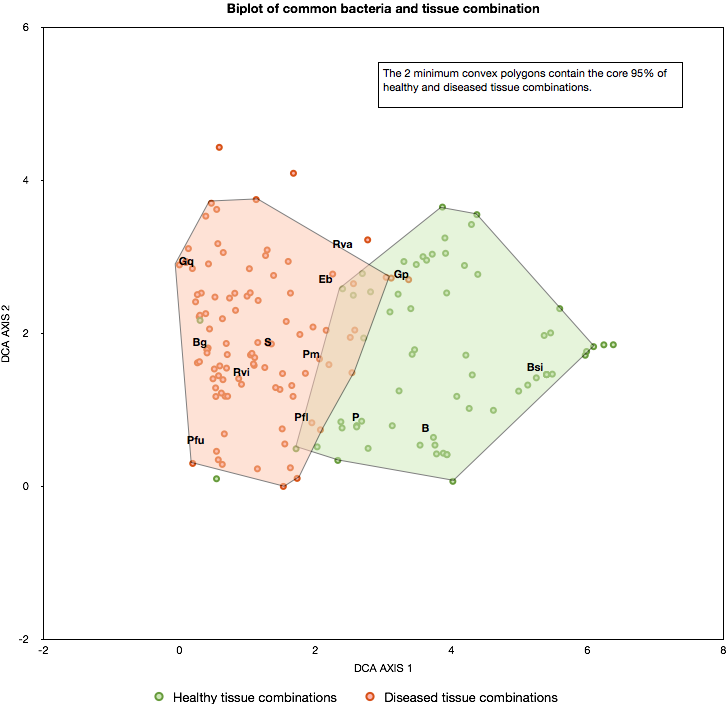

Supplement: Supplementary Figure S5 [file ismej2017170x6.docx]

**
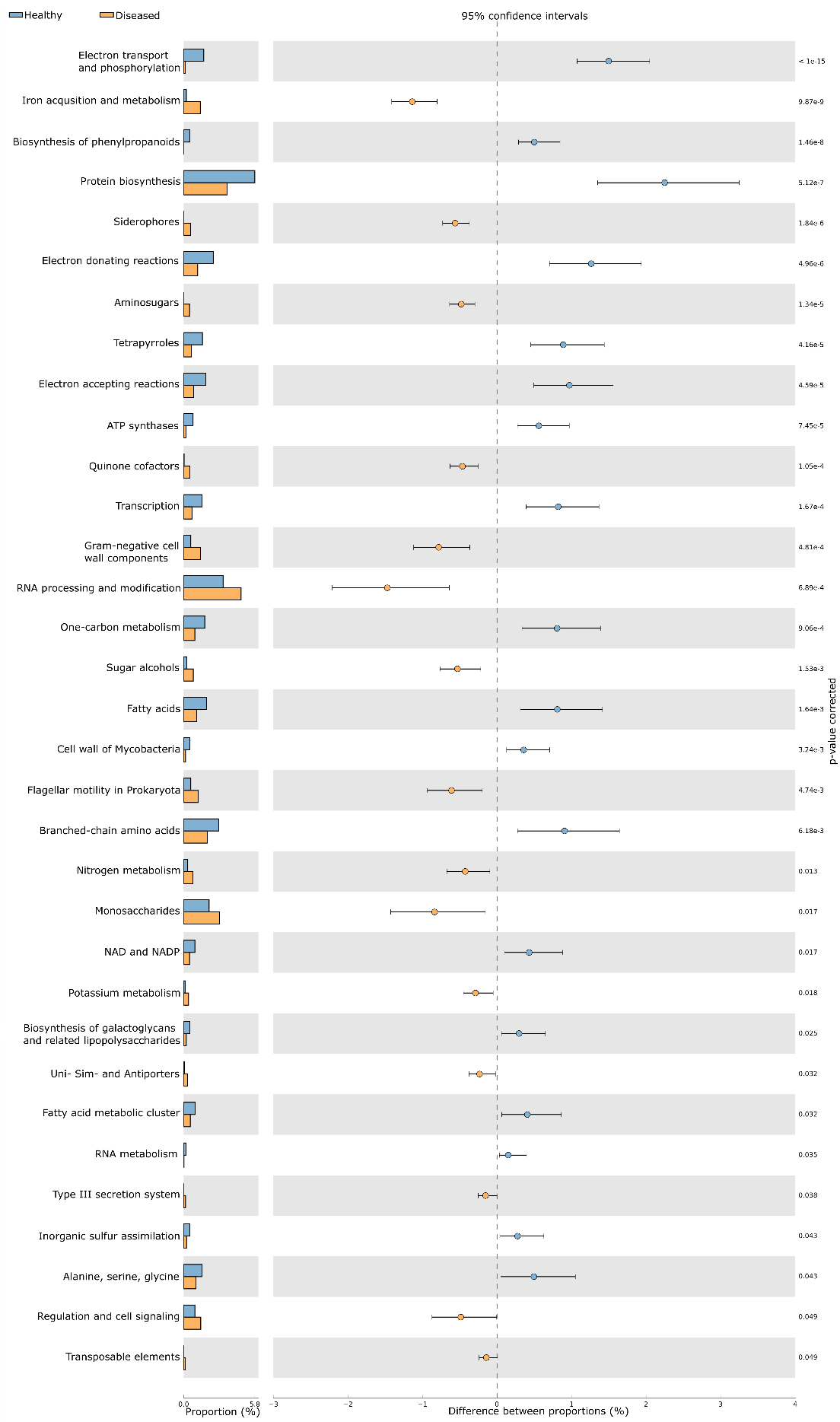
**

Supplement: Supplementary Figure S6 [file ismej2017170x7.docx]

**
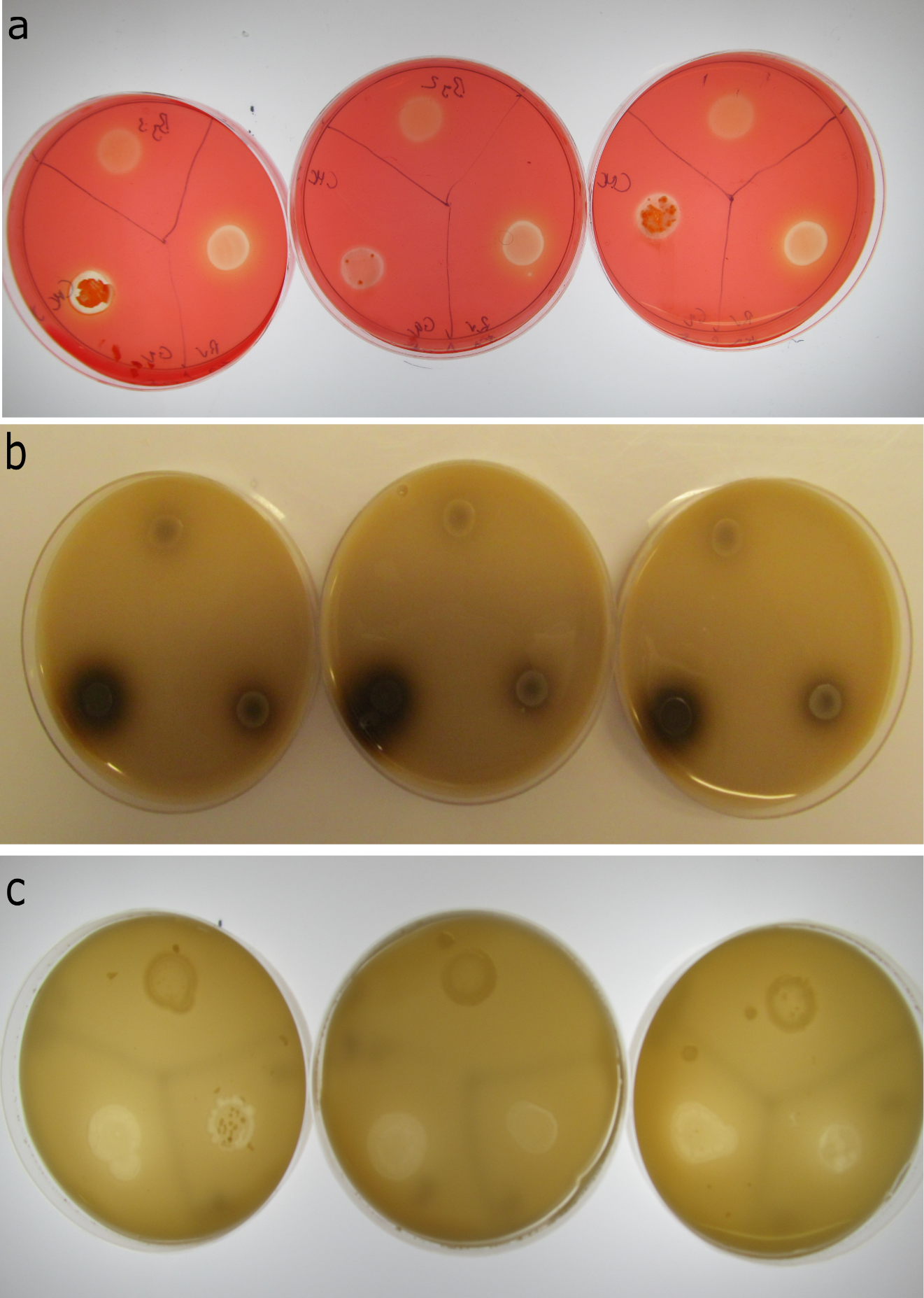
**

Supplement: Supplementary Figure S7 [file ismej2017170x8.docx]
