# Supplementary material for: Microbiome and infectivity studies reveal complex polyspecies tree disease in Acute Oak Decline
Source: ISME J. 2017 Oct 13;12(2):386–99. doi: 10.1038/ismej.2017.170 (PMC5776452; doi:10.1038/ismej.2017.170)
Supplement: Supplementary Methods and Figure Legends [file ismej2017170x9.docx]

**Supplementary Information**

**Supplementary Methods**

**Polysaccharide degradation tests:** Genome annotations of selected PCWDEs (plant cell wall degrading enzymes) encoded within *Brenneria goodwinii* FRB141, *Gibbsiella quercinecans* FRB97, and *Rahnella victoriana* BRK18a were validated phenotypically on polysaccharide substrates to qualitatively test for degradation (Supplementary Figure S7). Three biological replicates of each species were incubated overnight in nutrient broth (Oxoid) at 28˚C at 150 rpm and used as inocula for replicate polysaccharide degradation tests. For polysaccharide degradation tests, nutrient agar (Oxoid) was supplemented with three different polysaccharide substrates; 0.5% (w/v) carboxymethylcellulose (CMC) (Oxoid), 0.5% (w/v) tannic acid (TA) (Sigma), or 0.25% (w/v) polygalacturonic acid (PGA) (Sigma). Replicate broth cultures of each bacterial species were pipetted onto agar plates in 50 µl volumes and incubated at 28˚C. TA plates were visually inspected after two days for signs of degradation, which was visible as a brown/black discolouration surrounding the colony. After 5 days, CMC plates were flooded with 0.1% Congo red dye for 15 mins, followed by destaining by two washes with 1 M NaCl (sodium chloride) on a shaking incubator (50 rpm) for 15 mins at room temperature. Agar plates were visualised for cellulolytic activity, visible as a zone of clearing around the bacterial culture (Farrow and Arnold, 2011). After 11 days, PGA plates were stained with 1% cetyl trimethylammonium bromide (CTAB) and incubated overnight at room temperature, shaking gently at 50 rpm. Polygalacturonase (PG) activity was visible as a translucent halo around the bacterial colony (Cesbron *et al.*, 2015).

**Bioinformatic analysis:** General bioinformatic analyses were carried out on a locally installed Bio-Linux 8 workstation (Field *et al.*, 2006) and applications requiring high computing power were undertaken on the High Performance Computing (HPC) Wales supercomputing network.

**Genome assembly:** *de novo* genome assembly was performed on an Amazon EC2 image using the hierarchical genome assembly 3 (HGAP3) workflow (Chin *et al.*, 2013), incorporating the CELERA assembler. Resultant assemblies produced complete circular contigs for the *Gibbsiella quercinecans* FRB97 (T) and *Brenneria goodwinii* FRB141 (T) genomes, with average coverages of 400x and 393x, respectively. The assemblies were finished using Quiver consensus polisher, giving mean confidence values (QV) of 49 for both genomes. The *Rahnella victoriana* BRK18a (T) genome was assembled at the Centre for Genomic Research (CGR), University of Liverpool. HGAP3 assembled the genome into two contigs, which had an average sequencing coverage of 145x and 129x. The assembly was finished using Quiver consensus polisher, giving both contigs mean confidence values (QV) of 49.

**Genome annotation:** Whole genome general annotations were automatically generated using the Prokka annotation pipeline v1.11 (Seemann, 2014) and the RAST online annotation server (Aziz *et al.*, 2008). Prokka annotations were used as input to search for CAZymes, which were automatically identified using the dbCAN online server (Yin *et al.*, 2012). Type III, IV and VI secretion systems were annotated using T346 hunter (Martínez-García *et al.*, 2015). Whole genomes were displayed using the genome visualisation aesthetic Circos (Krzywinski *et al.*, 2009). Circos input data was generated using Bowtie2 v2.2.4 (Langmead and Salzberg, 2012), BEDTools v2.17.0 (Quinlan and Hall, 2010) and SAMtools v1.2 (Li *et al.*, 2009).

**Genome metrics:** Whole genome Pacific Biosciences RSII HGAP3 assemblies of *Gibbsiella quercinecans* FRB97 (T), *Brenneria goodwinii* FRB141(T), and *Rahnella victoriana* BRK18a (T), produced 1 contig for *G. quercinecans* FRB97 and *B. goodwinii* FRB141, and 2 contigs for *R. victoriana* BRK18a with N_50_ values of 5,548,506, 5,395,301, and 4,856,713, respectively (Supplementary Table S8). Genome assemblies were deposited in NCBI with accession numbers CP014136 (*G. quercinecans* FRB97), CP014137 (*B. goodwinii* FRB141) and MAEN00000000 (*R. victoriana* BRK18a).

**Metagenomic DNA extraction and metagenome sequencing of 4 healthy and 11 diseased oak trees:** Prior to nucleic acid extraction, tissue was frozen in liquid nitrogen and ground with a pestle in a mortar to homogenize the tissue. For samples AT1, AT7, AT8, AT9, RW1, RW2 and RW3, replicate (n=3) 0.6 g samples of homogenized tissue were weighed for extraction and DNA was extracted in triplicate using the Power Soil DNA Isolation kit (MoBio) and the replicates pooled. For samples AT2, AT3, AT4, AT5, AT6, ROW1, ROW2 and ROW3 DNA was extracted from approximately 50 mg of sample tissue using the DNeasy Plant Mini kit (Qiagen). The DNA for each sample was subjected to oak DNA depletion using the NEBNext® Microbiome DNA Enrichment Kit (New England Biolabs), with the exception of sample AT1, which was not enriched. Samples AT1, AT7, AT8, AT9, RW1, RW2 and RW3 were purified using Agencourt AMPure XP (Beckman Coulter) according to the manufacturer’s protocol, samples AT2, AT3, AT4, AT5, AT6, ROW1, ROW2 and ROW3 were purified and further concentrated using Genomic DNA Clean and Concentrator kit (Zymo research), according to the manufacturer’s instructions. DNA from all samples was quantified using a Qubit fluorometer (Thermo Fisher). Libraries for sequencing were prepared from samples using the Nextera XT Library preparation kit (Illumina), and sequenced using 2x100bp (samples AT1, AT7, AT8, AT9, RW1, RW2 and RW3) or 2x125 bp (samples AT2, AT3, AT4, AT5, AT6, ROW1, ROW2 and ROW3) paired-end sequencing on the Illumina HiSeq platform. Reads were trimmed using first Cutadapt v1.2.1 (Martin, 2011) with the option -O 3 and additionally Sickle v1.2.00 (Joshi and Fass, 2011) with a minimum quality score of 20.

**Statistical analyses of log lesion areas**

All lesion area analyses were conducted using the statistical package R (R Core Team, 2016); data were analysed using mixed-effects models in the lme4 package (Bates *et al.*, 2015). Random effects were assigned to account for the nesting within the data, with log nested within tree, and tree nested within experimental data set (2014 wound and non-wound, 2015, field data); given that lesion measurements were conducted following the same methodology across all trials, it was deemed reasonable to conduct the analysis in this way, with experimental data sets being treated as blocks, separated in both time and space: residual analysis (normally-distributed, homoscedastic residuals) supported this approach.. Bacteria x *A. biguttatus* combinations were included as fixed effects within the models. Lesion areas were standardised to normalise the residuals within the mixed-effects model, using an inverse transformation (*c*/(area + *c*), where *c* represents a fixed constant). Post-hoc tests were performed on the best-fit models to compare each treatment to the control data, using the appropriate adjustments for critical values (“dunnettx”, an approximation of the Dunnett adjustment) (Lenth, 2015; Graves *et al.*, 2015).

Back-isolations either yielded (a) no bacterial isolates (failed); (b) the organism(s) initially inoculated into the log (fulfilment of Koch’s postulates); (c) species of bacteria which the treatment inoculum did not contain (contamination). Koch’s postulates and contamination data were used to conduct two separate statistical analyses:

1. **Bacterial positive back-isolation**. For each bacterial species, the proportion of positive back isolations was calculated, both for where the species was included within the treatment and when it was not (i.e. contamination), and tested to see whether these were significantly different.
2. ***A. biguttatus* contamination.** For all treatments where *A. biguttatus* eggs or larvae were present, the proportion of contaminated samples was calculated, to test whether contamination of specific bacteria species was more likely in the presence of *A. biguttatus*.

All analyses were conducted in R (R Core Team, 2016); data were analysed using generalised mixed-effects models (Bates *et al.*, 2015), with the same random effects structure as for the lesion area analyses. Responses were binomial, with negative or positive back-isolation coded as 0 or 1. A binomial distribution with logit link function was applied in each case. The significance of factors was determined based on the Wald chi-square test statistics from the analysis of deviance, using the car package in R (Fox and Weisberg, 2011), with proportions calculated from post hoc tests in R (Lenth, 2015; Graves *et al.*, 2015) on the best-fit models.

**Figure legends**

**S 1 Location of oak sampling sites.** The number of healthy and diseased oaks sampled are shown for each location, the proportion of healthy oaks sampled are shown in light green and diseased oaks are red. The size of the pie charts is relative to the number oaks sampled. The locations shown have been adjusted to ensure pie charts do not overlap. Individual sites are numbered with full information is provided in Supplementary Table S2.

**S 2 Circular representation of *Pectobacterium carotovorum* subsp*. carotovorum* PC1 genome, and aligned homologous metatranscripts and metagenomic coding domains.** *Pectobacterium carotovorum* subsp*. carotovorum* PC1 is a canonical plant pathogen which is a member of the *Pectobacteriaceae* and was identified sporadically at low relative abundance from metagenomic libraries extracted from diseased tissue (Supplementary Table S4). However, P. *carotovorum* subsp*. carotovorum* PC1was not isolated from lesion tissue and despite being present at low relative abundance in metagenome samples we expect this was due to homologous alignment to conserved genes and an artefact of the alignment process. Therefore, it was selected as a control to measure the stringency of metagenome and metatranscriptome alignment. From outside to inside, circles represent: (1) Assembled P. *carotovorum* subsp*. carotovorum* PC1 genome, outermost (orange) circle, with encoded secretion systems annotated at their genomic loci. (2) Metatranscriptome heatmap. Alignment of two combined metatranscriptomes recovered from a necrotic lesion of an Acute Oak Decline (AOD) affected tree, against P. *carotovorum* subsp*. carotovorum* PC1. Blue saturation represents increasing transcript alignments. (3-9) Seven metagenomes from necrotic lesions on AOD affected trees and one healthy tree (metagenomes were extracted from two sites, Attingham and Runs Wood), were aligned through their coding domains to homologous regions in the P. *carotovorum* subsp*. carotovorum* PC1 genome. (3) Attingham healthy (AT1) aligned metagenome coding domains (light purple). (4) Attingham diseased (AT7) aligned metagenome coding domains (aqua). (5) Attingham diseased (AT8) aligned metagenome coding domains (blue). (6) Attingham diseased (AT9) aligned metagenome coding domains (orange). (7) Runs Wood diseased (RW1) aligned metagenome coding domains (green). (8) Runs Wood diseased (RW2) aligned metagenome coding domains (pink). (9) Runs Wood diseased (RW3) aligned metagenome coding domains (grey). (10) G+C content across the P. *carotovorum* subsp*. carotovorum* PC1 genome. (11) G+C skew across the P. *carotovorum* subsp*. carotovorum* PC1 genome.

S 3 Circular representation of *Paenibacillus polymyxa* SC2 genome, and aligned homologous metatranscripts and metagenomic coding domains. *Paenibacillus polymyxa* SC2 was identified within metagenomic samples at low relative abundance within healthy oak trees and diseased Acute Oak Decline (AOD) lesions (Supplementary Table S4). Therefore, a finished genome was downloaded from NCBI, and selected as a control to test the stringency of metagenome and metatranscriptome alignment. From outside to inside, circles represent: (1) Assembled *P. polymyxa* SC2 genome, outermost (orange) circle, with encoded secretion systems annotated at their genomic loci. (2) Metatranscriptome heatmap. Alignment of two combined metatranscriptomes recovered from a necrotic lesion of an AOD affected tree, against *P. polymyxa* SC2. Blue saturation represents increasing transcript alignments. (3-9) Seven metagenomes from necrotic lesions on AOD affected trees and one healthy tree (metagenomes were extracted from two sites, Attingham and Runs Wood), were aligned through their coding domains to homologous regions in the *P. polymyxa* SC2 genome. (3) Attingham healthy (AT1) aligned metagenome coding domains (light purple). (4) Attingham diseased (AT7) aligned metagenome coding domains (aqua). (5) Attingham diseased (AT8) aligned metagenome coding domains (blue). (6) Attingham diseased (AT9) aligned metagenome coding domains (orange). (7) Runs Wood diseased (RW1) aligned metagenome coding domains (green). (8) Runs Wood diseased (RW2) aligned metagenome coding domains (pink). (9) Runs Wood diseased (RW3) aligned metagenome coding domains (grey). (10) G+C content across the *P. polymyxa* SC2 genome. (11) G+C skew across the *P. polymyxa* SC2 genome.

**S 4** **Gene coverage of transcripts recovered from two necrotic lesions on Acute Oak Decline affected trees, against virulence genes encoded within *Brenneria goodwinii* FRB141, *Gibbsiella quercinecans* FRB97 and *Rahnella victoriana* BRK18a genomes.** Gene categories are represented by the following colors, red - plant cell wall degrading enzymes (PCWDEs), purple - general secretory pathway (GSP), yellow - type II secretion system (T2SS), blue - type III secretion system (T3SS), pink - type III secretion system effectors (T3SS effectors), and green - global regulators (GR).

**S 5 Detrended correspondence analysis of bacteria isolated from Acute Oak Decline diseased and healthy oak tissue.** Bacteria were grouped according to the tissue type from which they were isolated. The red and green shapes represent distinct ecological communities. Bacteria isolated from healthy tissue are in the green shape and diseased tissue within the red shape. N.B. Some bacteria were commonly isolated from healthy and diseased tissue, represented here in the overlap between healthy and diseased. B = *Bacillus* sp.; Bg = *Brenneria goodwinii*; Bsi = *Bacillus simplex*; Eb = *Erwinia billingiae*; Gp = Gram positive bacteria; Gq = *Gibbsiella quercinecans*; P = *Pseudomonas* sp.; Pfl – *Pseudomonas fluorescens*; Pfu = *Pseudomonas fulva*; Pm = *Pseudomonas marginalis;* Rva = *Rahnella* *variigena*; Rvi = *Rahnella* *victoriana*; S = *Stenotrophomonas* sp.

S 6 Functional metagenomic profile of Acute Oak Decline diseased and healthy oak tissue. Functional annotations were generated from MG-RAST level 2 data, and compared in STAMP, using a G-test with Yates’ correction to compare diseased and healthy profiles, and the Newcombe-Wilson method to calculate confidence intervals.

**S 7 Enzyme activity of *Brenneria goodwinii* FRB141, *Gibbsiella quercinecans* FRB97 and *Rahnella victoriana* BRK18a on polysaccharides analogous to those found in oak tissue.** Each Petri dish contains; ***Brenneria goodwinii* FRB141** (top), ***Gibbsiella quercinecans* FRB97** (bottom left) and ***Rahnella victoriana*** (bottom right). Bacteria were grown on polysaccharide substrates; (a) carboxymethylcellulose (5 day incubation); (b) tannic acid (2 day incubation); (c) polygalacturonic acid (11 day incubation). Degradation was visible for *G. quercinecans* and *R. victoriana* on all substrates, i.e. (a) white halo extending from bacterial colony, (b) brown/black plaque extending from bacterial colony, and (c) translucent halo extending from bacterial colony. At the specified incubation period *B. goodwinii* had no visible degradative activity on any of the substrates.

**References for Supplemental Data**

Aziz RK, Bartels D, Best AA, DeJongh M, Disz T, Edwards RA, *et al.* (2008). The RAST server: rapid annotations using subsystems technology. *BMC Genomics* **9**: 75.

Bates D, Mächler M, Bolker B, Walker S. (2015). Fitting linear mixed-effects models using lme4. *J Stat Softw* **67**: 1–48.

Chin C-S, Alexander DH, Marks P, Klammer AA, Drake J, Heiner C, *et al.* (2013). Nonhybrid, finished microbial genome assemblies from long-read SMRT sequencing data. *Nat Methods* **10**: 563–569.

Field D, Tiwari B, Booth T, Houten S, Swan D, Bertrand N, *et al.* (2006). Open software for biologists: from famine to feast. *Nat Biotechnol* **24**: 801–803.

Graves S, Piepho H-P, Selzar L, Dorai-Raj S. (2015). multcompView: Visualizations of Paired Comparisons. https://cran.r-project.org/package=multcompView.

Joshi N, Fass J. (2011). Sickle. A sliding-window, adaptive, quality-based trimming tool for FastQ files. https:// github.com/najoshi/sickle

Krzywinski M, Schein J, Birol I, Connors J, Gascoyne R, Horsman D, *et al.* (2009). Circos: an information esthetic for comparative genomics. *Genome Res* **19**: 1639–1645.

Langmead B, Salzberg SL. (2012). Fast gapped-read alignment with Bowtie 2. *Nat Methods* **9**: 357–9.

Lenth R. (2015). lsmeans: Least-Squares Means. https://cran.r-project.org/package=lsmeans.

Li H, Handsaker B, Wysoker A, Fennell T, Ruan J, Homer N, *et al.* (2009). The sequence alignment/map format and SAMtools. *Bioinformatics* **25**: 2078–2079.

Martin M. (2011). Cutadapt removes adapter sequences from high-throughput sequencing reads. *EMBnet.journal* **17**: 10-12.

Martínez-García PM, Ramos C, Rodríguez-Palenzuela P. (2015). T346Hunter: A novel web-based tool for the prediction of type III, type IV and type VI secretion systems in bacterial genomes. *PLoS One* **10**: e0119317.

Quinlan AR, Hall IM. (2010). BEDTools: A flexible suite of utilities for comparing genomic features. *Bioinformatics* **26**: 841–842.

R Core Team. (2016). R: A language and environment for statistical computing. R foundation for statistical computing. https://www.r-project.org/.

Seemann T. (2014). Prokka: rapid prokaryotic genome annotation. *Bioinformatics* **30**: 2068–2069.

Fox J, Weisberg S. (2011). An R Companion to Applied Regression. 2nd ed. SAGE: California.

Yin Y, Mao X, Yang J, Chen X, Mao F, Xu Y. (2012). DbCAN: A web resource for automated carbohydrate-active enzyme annotation. *Nucleic Acids Res* **40**: 445–451.
