# Supplementary material for: Microbiome and infectivity studies reveal complex polyspecies tree disease in Acute Oak Decline
Source: ISME J. 2017 Oct 13;12(2):386–99. doi: 10.1038/ismej.2017.170 (PMC5776452; doi:10.1038/ismej.2017.170)
Supplement: Supplementary Table 1 [file ismej2017170x10.docx]

**Supplementary Table 1**. **Annual log inoculation test treatments using single and multiple bacterial species, with or without the eggs of *Agrilus biguttatus***.

| **Inoculation treatment** | **Year and treatments used** | | | | **Inoculation treatment with *Ab*^d^ eggs** | **Year and treatments used** | | | |
| --- | --- | --- | --- | --- | --- | --- | --- | --- | --- |
|  | **2014 W^a^** | **2014 NW^b^** | **2015 W** | **2015 FT^c^** |  | **2014 W^a^** | **2014 NW^b^** | **2015 W** | **2015 FT^c^** |
| ^e^*Bg* | Y | N | Y | Y | *Bg* + *Ab* eggs | Y | Y | Y | Y |
| ^f^*Gq* | Y | N | Y | Y | *Gq* + *Ab* eggs | Y | Y | Y | Y |
| ^g^*Lqb* | N | N | Y | Y | *Lqb*+ *Ab* eggs | N | N | Y | Y |
| ^h^*Rvi* | N | N | Y | Y | *Rvi* + *Ab* eggs | N | N | Y | Y |
| ^i^*Rva* | N | N | Y | Y | *Rva* + *Ab* eggs | N | N | Y | Y |
| Water (control) | Y | N | Y | Y | *Ab* egg only | Y | Y | Y | Y |
| ^j^*Eb* | N | N | Y | Y | *Eb* + *Ab* eggs | N | N | Y | Y |
| *Bg* + *Gq* | Y | N | Y | Y | *Bg* + *Gq* + *Ab* eggs | Y | Y | Y | Y |
| *Bg* + *Gq + Lqb* | N | N | Y | Y | *Bg* + *Gq + Lqb*+ *Ab* eggs | N | N | Y | Y |
| *Bg* + *Gq + Rvi* | N | N | Y | Y | *Bg* + *Gq + Rvi* + *Ab* eggs | N | N | Y | Y |
| *Bg* + *Gq + Rva* | N | N | Y | Y | *Bg* + *Gq + Rva* + *Ab* eggs | N | N | Y | Y |
| *Bg* + *Gq + Lqb + Rvi* + *Rva* | N | N | Y | Y | *Bg* + *Gq + Lqb + Rvi* + *Rva* + *Ab* eggs | N | N | Y | Y |

^a^ W = wound log inoculations

^b^ NW = non-wound log inoculations

^c^ FT = field trial

^d^ Ab = *Agrilus biguttatus*

^e^ Bg = *Brenneria goodwinii*

^f^ Gq = *Gibbsiella quercinecans*

^g^ Lqb = *Lonsdalea quercina* ssp *britanica*

^h^ Rvi = *Rahnella victoriana*

^i^ Rva = *Rahnella variigena*

^j^ Eb = *Erwinia billingiae*
