# Supplementary material for: Microbiome and infectivity studies reveal complex polyspecies tree disease in Acute Oak Decline
Source: ISME J. 2017 Oct 13;12(2):386–99. doi: 10.1038/ismej.2017.170 (PMC5776452; doi:10.1038/ismej.2017.170)
Supplement: Supplementary Table 2 [file ismej2017170x11.docx]

**Supplementary Table 2. Forest and woodland sites used for bacterial isolation from Acute Oak Decline diseased and healthy oak trees.** Microbiome samples for metagenomic analysis were gathered at three of these sites, two sites in Attingham park were sampled on two dates: Samples AT1, AT7, AT8 and AT9 were collected from OS Eastings 356033, Northings 310372, in November 2013; and samples AT2, AT3, AT4, AT5 and AT6 at OS Eastings 356033, Northings 310372, in June 2015. Samples RW1, RW2 and RW3 were collected from Runs Wood in February 2014 (OS Eastings 563207, Northings 310858), and samples ROW1, ROW2 and ROW3 were collected from Ross-on-Wye (OS Eastings 357887, Northings 221731), in June 2015. Ordinance survey co-ordinates are provided.

| Ref. to  Figure S1 | Site | Number  Healthy | Number  Diseased | Total | Longitude | Latitude |
| --- | --- | --- | --- | --- | --- | --- |
| 1 | Attingham Healthy | 2 | 4 | 6 | -2.669 | 52.685 |
| 2 | Bisham, Park Wood | 1 | 1 | 2 | -0.770 | 51.549 |
| 3 | Bovingdon Hall | 1 | 2 | 3 | 0.554 | 51.925 |
| 4 | Brockhampton | 0 | 2 | 2 | -2.455 | 52.201 |
| 5 | Bungate Wood | 1 | 2 | 3 | 0.657 | 51.892 |
| 6 | Coleshill Estate | 0 | 1 | 1 | -1.659 | 51.631 |
| 7 | Crumblands Plantation | 3 | 0 | 3 | -2.761 | 51.720 |
| 8 | Delamere | 0 | 1 | 1 | -2.678 | 53.229 |
| 9 | Forest of Dean | 0 | 1 | 1 | -2.573 | 51.801 |
| 10 | Great Monks Wood | 1 | 2 | 3 | 0.645 | 51.896 |
| 11 | Gorse Covert | 0 | 1 | 1 | -1.243 | 52.781 |
| 12 | Hatchlands | 1 | 1 | 2 | -0.472 | 51.258 |
| 13 | Hoddesdon Park | 0 | 2 | 2 | -0.049 | 51.756 |
| 14 | Malvern | 1 | 2 | 3 | -2.308 | 52.085 |
| 15 | Micheldever Forest | 3 | 0 | 3 | -1.239 | 51.137 |
| 16 | Oakhill Estate | 0 | 2 | 2 | 0.248 | 51.215 |
| 17 | Old Steads Forest | 3 | 0 | 3 | -1.181 | 54.222 |
| 18 | Outwood | 0 | 2 | 5 | -1.239 | 52.739 |
| 19 | Owston Wood | 3 | 0 | 3 | -0.850 | 52.650 |
| 20 | Pirton | 2 | 1 | 3 | -2.185 | 52.115 |
| 21 | Ross-on-Wye | 1 | 2 | 3 | -2.613 | 51.892 |
| 22 | Runs Wood | 1 | 3 | 4 | 0.412 | 52.671 |
| 23 | Send | 1 | 1 | 2 | -0.529 | 51.288 |
| 24 | Spinneywood | 0 | 1 | 1 | -1.247 | 52.768 |
| 25 | Sotterley | 0 | 2 | 2 | 1.616 | 52.409 |
| 26 | Staple Park Wood | 3 | 0 | 3 | -3.075 | 50.950 |
| 27 | Stratfield Brake | 1 | 2 | 3 | -1.285 | 51.804 |
| 28 | Winding Wood | 0 | 1 | 1 | 0.846 | 52.053 |
| 29 | Wyre | 0 | 1 | 1 | -2.356 | 52.380 |
