# Supplementary material for: Microbiome and infectivity studies reveal complex polyspecies tree disease in Acute Oak Decline
Source: ISME J. 2017 Oct 13;12(2):386–99. doi: 10.1038/ismej.2017.170 (PMC5776452; doi:10.1038/ismej.2017.170)
Supplement: Supplementary Table 3 [file ismej2017170x12.docx]

| Library | Site | Health status | Sequencing type | Accession number/Sequence read archive | BioSample | BioProject |
| --- | --- | --- | --- | --- | --- | --- |
| AT1 | Attingham | Healthy | Metagenome | SRP076579 | SAMN05172600 | PRJNA323828 |
| AT2 | Attingham | Healthy | Metagenome | SRP076012 | SAMN05150009 | PRJNA321868 |
| AT3 | Attingham | Healthy | Metagenome | SRP076012 | SAMN05150010 | PRJNA321868 |
| AT4 | Attingham | Healthy | Metagenome | SRP076012 | SAMN05150011 | PRJNA321868 |
| AT5 | Attingham | Diseased | Metagenome | SRP076012 | SAMN05150012 | PRJNA321868 |
| AT6 | Attingham | Diseased | Metagenome | SRP076012 | SAMN05150013 | PRJNA321868 |
| AT7 | Attingham | Diseased | Metagenome | SRP076579 | SAMN05150018 | PRJNA323828 |
| AT8 | Attingham | Diseased | Metagenome | SRP076579 | SAMN05150019 | PRJNA323828 |
| AT9 | Attingham | Diseased | Metagenome | SRP076579 | SAMN05150020 | PRJNA323828 |
| AT11 | Attingham | Diseased | Metatranscriptome | SRP076579 | SAMN05172600 | PRJNA323828 |
| AT12 | Attingham | Diseased | Metatranscriptome | SRP076579 | SAMN05172601 | PRJNA323828 |
| RW1 | Runs Wood | Diseased | Metagenome | SRP076579 | SAMN05150021 | PRJNA323828 |
| RW2 | Runs Wood | Diseased | Metagenome | SRP076579 | SAMN05150022 | PRJNA323828 |
| RW3 | Runs Wood | Diseased | Metagenome | SRP076579 | SAMN05150023 | PRJNA323828 |
| ROW1 | Ross-on-Wye | Diseased | Metagenome | SRP076012 | SAMN05150014 | PRJNA321868 |
| ROW2 | Ross-on-Wye | Diseased | Metagenome | SRP076012 | SAMN05150015 | PRJNA321868 |
| ROW3 | Ross-on-Wye | Diseased | Metagenome | SRP076012 | SAMN05150016 | PRJNA321868 |
| *Gibbsiella quercinecans* FRB97 | - | - | Genome | SRP076579  *CP014136 | SAMN04388492 | PRJNA323828 |
| *Brenneria goodwinii* FRB141 | - | - | Genome | SRP076579  *CP014137 | SAMN04388496 | PRJNA323828 |
| *Rahnella victoriana* BRK18a | - | - | Genome | SRP076579  *MAEN00000000 | SAMN05249967 | PRJNA323828 |

**Supplementary Table 3. Accession numbers for genomic, metagenomic and metatranscriptomic libraries.** All data was submitted to the National Centre for Biotechnology Information. *Genome assembly
