# Supplementary material for: Microbiome and infectivity studies reveal complex polyspecies tree disease in Acute Oak Decline
Source: ISME J. 2017 Oct 13;12(2):386–99. doi: 10.1038/ismej.2017.170 (PMC5776452; doi:10.1038/ismej.2017.170)
Supplement: Supplementary Table 4 [file ismej2017170x13.docx]

**Supplementary Table 4. Alignment of metagenome coding domains to the genomes of *Brenneria goodwinii* FRB141 (T), *Gibbsiella quercinecans* FRB97 (T), and *Rahnella victoriana* BRK18a (T) and two control genomes, *Pectobacterium carotovorum* subsp. *carotovorum* PC1, a known plant pathogen, and the endophyte, *Paenibacillus polymyxa* SC2.** To assess the abundance of *Brenneria goodwinii* (*Bg*)**,** *Gibbsiella quercinecans* (*Gq*), and *Rahnella victoriana* (*Rv*) in the Acute Oak Decline (AOD) lesion metagenome, alignments were made between assembled metagenomic libraries from AOD diseased and a healthy tree (AT1), and assembled bacterial coding domains of *Bg*, *Gq* and *Rv* BRK18a (T). In addition, to assess the veracity of our alignment approach, we tested two additional control genomes; *Pectobacterium carotovorum* subsp. *carotovorum* PC1 (PC1) is a well-characterised bacterial phytopathogen that has not previously been associated with oak. This genome would therefore not be expected to be present within AOD lesion metagenomes, and therefore provides an indication of the potential level of alignment matches from homologous genes found within other members of the *Pectobacteriaceae* and *Enterobacteriaceae*. As table S5 demonstrates, only a small proportion of metagenome coding domains (44-183) mapped with PC1, suggesting that the greater proportion of matches that occur between *Gq*, *Bg* and *Rv* reflect the actual presence and abundance of those species in AOD lesion metagenomes, rather than them representing metagenome coding domains from other *Enterobacteriaceae* that have been falsely attributed to our focal species (*Bg, Gq* and *Rv*). *Paenibacillus polymyxa* SC2 is a putative endophyte found at low relative abundance within symptomatic and healthy oak tissue and was included as a control to ensure that the high numbers of metagenome coding domains mapped to *Gq* FRB97, *Bg* FRB141 and *Rv* BRK18a are accurate and would not occur in the same way for other *Pectobacteriaceae* and *Enterobacteriaceae* that are known to also be present in AOD lesions. The low numbers of coding domains that map against *P. polymyxa* SC2 (0-8) again supports the veracity of our alignment approach, and the abundance of *Bg, Gq* and *Rv* in AOD lesion tissue.

| Metagenome | Total genes | *G. quercinecans* FRB97 | *B. goodwinii* FRB141 | *R. victoriana* BRK18a | *P. polymyxa* SC2 | *P. carotovorum* subsp. *carotovorum* PC1 |
| --- | --- | --- | --- | --- | --- | --- |
| AT1 | 58146 | 1 | 2 | 2 | 0 | 0 |
| AT7 | 216140 | 2507 | 56 | 47 | 1 | 44 |
| AT8 | 192147 | 1493 | 3838 | 1914 | 6 | 110 |
| AT9 | 227496 | 72 | 1001 | 38 | 0 | 178 |
| RW1 | 211149 | 61 | 4181 | 49 | 1 | 183 |
| RW2 | 195109 | 23 | 428 | 256 | 5 | 51 |
| RW3 | 196863 | 993 | 3846 | 72 | 8 | 165 |
