# Supplementary material for: Microbiome and infectivity studies reveal complex polyspecies tree disease in Acute Oak Decline
Source: ISME J. 2017 Oct 13;12(2):386–99. doi: 10.1038/ismej.2017.170 (PMC5776452; doi:10.1038/ismej.2017.170)
Supplement: Supplementary Table 5 [file ismej2017170x14.docx]

**Supplementary Table 6. Occurrence of most commonly isolated bacterial taxa on sites and trees.**

| Taxon | Number of sites with bacteria: | | Number of trees with bacteria: | |
| --- | --- | --- | --- | --- |
|  | Healthy tissue | Symptomatic tissue | Healthy trees | Symptomatic trees |
| *Gibbsiella quercinecans* | 1/16 | 23/23 | 1/28 | 31/38 |
| *Brenneria goodwinii* | 2/16 | 15/23 | 2/28 | 23/38 |
| *Pseudomonas* sp. | 9/16 | 12/23 | 11/28 | 15/38 |
| Gram positive = other | 6/16 | 9/23 | 6/28 | 9/38 |
| *Erwinia billingiae* | 5/16 | 8/23 | 5/28 | 12/38 |
| *Rahnella victoriana* | 0/16 | 9/23 | 0/28 | 14/38 |
| *Bacillus* simplex | 9/16 | 0/23 | 17/28 | 0/38 |
| *Bacillus* sp. | 11/16 | 4/23 | 13/28 | 4/38 |
| *Pseudomonas fluorescens* | 2/16 | 10/23 | 2/28 | 12/38 |
| *Pseudomonas fulva*-like | 0/16 | 8/23 | 0/28 | 11/38 |
| *Stenotrophomonas* sp. | 1/16 | 7/23 | 1/28 | 9/38 |
| *Rahnella variigena* | 2/16 | 7/23 | 2/28 | 7/38 |
| *Pseudomonas marginalis* | 2/16 | 8/23 | 2/28 | 9/38 |
| *Lonsdalea quercina* ssp. *britanica* | 0/16 | 4/23 | 0/28 | 5/38 |
