# Supplementary material for: Microbiome and infectivity studies reveal complex polyspecies tree disease in Acute Oak Decline
Source: ISME J. 2017 Oct 13;12(2):386–99. doi: 10.1038/ismej.2017.170 (PMC5776452; doi:10.1038/ismej.2017.170)
Supplement: Supplementary Table 6 [file ismej2017170x15.docx]

**Supplementary Table 7. Significance of genera detected in healthy and symptomatic metagenome samples.** Welch's unequal variances *t*-tests of genera detected in metagenome datasets derived from healthy and diseased oak trees. ^a^Asymptotically F distributed.

|  | | Statistic^a^ | df1 | df2 | Sig. |
| --- | --- | --- | --- | --- | --- |
| *Periglandula* | Welch | 821.752 | 1 | 3.573 | .000 |
| *Burkholderia* | Welch | 23.221 | 1 | 3.011 | .017 |
| *Streptomyces* | Welch | 49.699 | 1 | 3.373 | .004 |
| *Aureimonas* | Welch | 21.779 | 1 | 3.019 | .018 |
| *Bacillus* | Welch | 13.394 | 1 | 3.009 | .035 |
| *Granulicella* | Welch | 1.895 | 1 | 3.004 | .262 |
| *Pseudomonas* | Welch | 1.075 | 1 | 11.057 | .322 |
| *Conexibacter* | Welch | 1.681 | 1 | 3.006 | .285 |
| *Valsa* | Welch | 186.320 | 1 | 3.084 | .001 |
| *Bradyrhizobium* | Welch | .197 | 1 | 3.371 | .684 |
| *Mycobacterium* | Welch | .046 | 1 | 3.832 | .841 |
| *Sphingomonas* | Welch | .001 | 1 | 8.482 | .981 |
| *Solirubrobacter* | Welch | 1.636 | 1 | 3.006 | .291 |
| *Terriglobus* | Welch | 1.800 | 1 | 3.004 | .272 |
| *Penicillium* | Welch | 11.869 | 1 | 3.012 | .041 |
| *Empedobacter* | Welch | 22.423 | 1 | 3.012 | .018 |
| *Salmonella* | Welch | .844 | 1 | 12.661 | .375 |
| *Brenneria* | Welch | 12.406 | 1 | 10.000 | .006 |
| *Paenibacillus* | Welch | 3.718 | 1 | 10.002 | .083 |
| *Rahnella* | Welch | 3.304 | 1 | 10.001 | .099 |
| *Serratia* | Welch | 3.222 | 1 | 10.009 | .103 |
| *Erwinia* | Welch | 1.952 | 1 | 10.000 | .193 |
| *Pelosinus* | Welch | 4.248 | 1 | 10.000 | .066 |
| *Microbacterium* | Welch | 7.190 | 1 | 10.389 | .022 |
| *Cellulomonas* | Welch | 6.009 | 1 | 10.273 | .034 |
| *Gibbsiella* | Welch | 4.741 | 1 | 10.316 | .054 |
| *Komagataella* | Welch | 3.617 | 1 | 10.000 | .086 |
| *Mesorhizobium* | Welch | .974 | 1 | 10.318 | .346 |
| *Nesterenkonia* | Welch | 3.064 | 1 | 10.001 | .111 |
| *Xanthomonas* | Welch | 8.992 | 1 | 10.645 | .013 |
| *Luteimonas* | Welch | 6.588 | 1 | 10.085 | .028 |
| *Arthrobacter* | Welch | 4.899 | 1 | 10.914 | .049 |
| *Halomonas* | Welch | 5.042 | 1 | 10.700 | .047 |
| *Stenotrophomonas* | Welch | 11.104 | 1 | 10.420 | .007 |
| *Sanguibacter* | Welch | 3.640 | 1 | 10.000 | .086 |
| *Devosia* | Welch | 3.951 | 1 | 10.752 | .073 |
| *Hymenobacter* | Welch | 1.221 | 1 | 10.063 | .295 |
| *Dysgonomonas* | Welch | 2.401 | 1 | 10.000 | .152 |
| *Proteiniphilum* | Welch | 3.007 | 1 | 10.001 | .114 |
| *Nocardiopsis* | Welch | 2.248 | 1 | 10.419 | .163 |
| *Paracoccus* | Welch | 5.382 | 1 | 11.588 | .039 |
| *Rhizobium* | Welch | 2.183 | 1 | 7.675 | .179 |
| *Escherichia* | Welch | 1.455 | 1 | 10.001 | .256 |
| *Lonsdalea* | Welch | 2.912 | 1 | 10.000 | .119 |
