# Supplementary material for: Microbiome and infectivity studies reveal complex polyspecies tree disease in Acute Oak Decline
Source: ISME J. 2017 Oct 13;12(2):386–99. doi: 10.1038/ismej.2017.170 (PMC5776452; doi:10.1038/ismej.2017.170)
Supplement: Supplementary Table 7 [file ismej2017170x16.docx]

Supplementary Table 8. Genome metrics of *Brenneria goodwinii* FRB141 (T), *Gibbsiella quercinecans* FRB97 (T), and *Rahnella victoriana* BRK18a (T) sequenced bacterial isolates. Bacteria were isolated from necrotic lesions of Acute Oak Decline affected trees.

| Organism | Sequencing platform | Assembler | Contigs | N_50_ (G+C content %) | No. of genes (Coding density %) | Chromosome size (bp) |
| --- | --- | --- | --- | --- | --- | --- |
| *Gibbsiella quercinecans* FRB97 | Pacific Biosciences RSII | HGAP3 | 1 | 5,548,506  (55.9) | 5126 (87.2) | 5,548,506 |
| *Brenneria goodwinii* FRB141 | Pacific Biosciences RSII | HGAP3 | 1 | 5,395,301  (53.1) | 4905 (86.3) | 5,395,301 |
| *Rahnella victoriana* BRK18a | Pacific Biosciences RSII | HGAP3 | 2 | 4,856,713  (53.5) | 5230 (88.9) | 5,563,295 |
