# Supplementary material for: Microbiome and infectivity studies reveal complex polyspecies tree disease in Acute Oak Decline
Source: ISME J. 2017 Oct 13;12(2):386–99. doi: 10.1038/ismej.2017.170 (PMC5776452; doi:10.1038/ismej.2017.170)
Supplement: Supplementary Table 8 [file ismej2017170x17.docx]

**Supplementary Table 9. Genomic loci of putative virulence genes.** Genes encoded within *Brenneria goodwinii* FRB141 (T), *Gibbsiella quercinecans* FRB97 (T), and *Rahnella victoriana* (T) BRK18a. PCWDE – plant cell wall degrading enzymes, T2SS – type II secretion system, T3SS – type III secretion system, T4SS – type IV secretion system.

| *Brenneria goodwinii* FRB141 | |  |  |  |
| --- | --- | --- | --- | --- |
| **PCWDE** |  |  |  |  |
| CAZy | EC | Enzyme name | Start | End |
| PL2 | 4.2.2.9 | Pectate disaccharide-lyase | 1893607 | 1895238 |
| PL3 | 4.2.2.2 | Pectate lyase | 1955948 | 1957357 |
| PL22 | 4.2.2.6 | Oligogalacturonate lyase | 1918944 | 1920110 |
| PL22 | 4.2.2.6 | Oligogalacturonate lyase | 2705052 | 2706344 |
| GH78 | 3.2.1.40 | α-L-rhamnosidase | 1293829 | 1295523 |
| GH36 | 3.2.1.22 | α-galactosidase | 1596206 | 1598329 |
| GH3 | 3.2.1.21 | β-glucosidase | 28527 | 30476 |
| GH3 | 3.2.1.21 | β-glucosidase | 1081804 | 1084110 |
| GH3 | 3.2.1.21 | β-glucosidase | 1231123 | 1232151 |
| GH3 | 3.2.1.21 | β-glucosidase | 3650290 | 3652491 |
| GH2 | 3.2.1.23 | β-galactosidase | 3196765 | 3199863 |
| GH1 | 3.2.1.86 | 6-phospho-β-glucosidase | 21146 | 22618 |
| GH1 | 3.2.1.86 | 6-phospho-β-glucosidase | 22692 | 24089 |
| GH1 | 3.2.1.86 | 6-phospho-β-glucosidase | 1292345 | 1293739 |
| GH1 | 3.2.1.86 | 6-phospho-β-glucosidase | 3557721 | 3559109 |
| GH1 | 3.2.1.86 | 6-phospho-β-glucosidase | 4301334 | 4302779 |
| GH1 | 3.2.1.86 | 6-phospho-β-glucosidase | 4948150 | 4949574 |
| GH5 | 3.2.1.4 | Cellulase | 1542654 | 1544144 |
| CE1 | 3.1.1.73 | Feruloyl esterase | 832390 | 834114 |
|  |  |  |  |  |
| **T3SS** |  |  |  |  |
| SctC |  |  | 1959915 | 1961951 |
| SctL |  |  | 1964956 | 1965561 |
| SctJ |  |  | 1966165 | 1966935 |
| SctI |  |  | 1966942 | 1967304 |
| SctW |  |  | 1972113 | 1973270 |
| SctV |  |  | 1973267 | 1975369 |
| SctD |  |  | 1975379 | 1976326 |
| SctN |  |  | 1976328 | 1977698 |
| SctQ |  |  | 1978646 | 1979728 |
| SctR |  |  | 1979725 | 1980378 |
| SctS |  |  | 1980386 | 1980640 |
| SctT |  |  | 1980656 | 1981453 |
| SctU |  |  | 1981464 | 1982540 |
| DsbA/E |  |  | 2999978 | 3000628 |
| DsbF |  |  | 3000915 | 3006419 |
| DsbA/E |  |  | 3006628 | 3007050 |
| YopJ |  |  | 4777750 | 4778814 |
| YopT |  |  | 3988757 | 3989768 |
| HopM1 |  |  | 4892530 | 4894635 |
| HopPto |  |  | 1722149 | 1724620 |
| HopPto |  |  | 1724617 | 1727613 |
| HopPto |  |  | 1727610 | 1729013 |
| HrpX |  |  | 1969597 | 1971063 |
|  |  |  |  |  |
| **Regulators** |  |  |  |  |
| RsmA |  |  | 301672 | 301857 |
| LuxS |  |  | 306131 | 306646 |
| GacA |  |  | 2264809 | 2266641 |
| RpoS |  |  | 178015 | 179007 |
| RsmB |  |  | 3071619 | 3071977 |
| KdgR |  |  | 1341592 | 1342386 |
| PhoP |  |  | 1935631 | 1936323 |
| PhoQ |  |  | 1936316 | 1937842 |
| VirF |  |  | 221039 | 221950 |
| XlyR |  |  | 4423762 | 4424946 |
| OmpR |  |  | 3625199 | 3625984 |
|  |  |  |  |  |
| *Gibbsiella quercinecans* FRB97 | |  |  |  |
| **PCWDE** |  |  |  |  |
| CAZy | EC | Enzyme name | Start | End |
| PL4 | 4.2.2.23 | Rhamnogalacturonan endolyase | 4763632 | 4765338 |
| PL22 | 4.2.2.6 | Oligogalacturonate lyase | 1192660 | 1193952 |
| PL22 | 4.2.2.6 | Oligogalacturonate lyase | 2499689 | 2500975 |
| GH78 | 3.2.1.40 | α-L-rhamnosidase | 1187035 | 1189665 |
| GH78 | 3.2.1.40 | α-L-rhamnosidase | 4655962 | 4657668 |
| GH78 | 3.2.1.40 | α-L-rhamnosidase | 4756055 | 4756747 |
| GH53 | 3.2.1.89 | Arabinogalactan endo-β-1,4-galactanase | 3021290 | 3022492 |
| GH3 | 3.2.1.21 | β-glucosidase | 204949 | 205989 |
| GH3 | 3.2.1.21 | β-glucosidase | 1110861 | 1113158 |
| GH3 | 3.2.1.21 | β-glucosidase | 5404947 | 5406908 |
| GH28 | 3.2.1.15 | Polygalacturonase | 1064677 | 1065930 |
| GH2 | 3.2.1.23 | β-galactosidase | 311950 | 315036 |
| GH1 | 3.2.1.86 | 6-phospho-β-glucosidase | 273119 | 274549 |
| GH1 | 3.2.1.86 | 6-phospho-β-glucosidase | 610972 | 612375 |
| GH1 | 3.2.1.86 | 6-phospho-β-glucosidase | 642629 | 644065 |
| GH1 | 3.2.1.86 | 6-phospho-β-glucosidase | 2416794 | 2418197 |
| GH30 | 3.2.1.45 | Glucosylceramidase | 4305197 | 4306531 |
| GH8 | 3.2.1.4 | Cellulase | 3817866 | 3818867 |
| GH42 | 3.2.1.23 | β-galactosidase | 3022519 | 3024576 |
|  |  |  |  |  |
| **T2SS** |  |  |  |  |
| OutC |  |  | 4831705 | 4832508 |
| OutD |  |  | 4832557 | 4834485 |
| OutE |  |  | 4834482 | 4835972 |
| OutF |  |  | 4835974 | 4837197 |
| OutG |  |  | 4837231 | 4837656 |
| OutH |  |  | 4837660 | 4838202 |
| OutI |  |  | 4838199 | 4838582 |
| OutJ |  |  | 4838579 | 4839178 |
| OutK |  |  | 4839178 | 4840125 |
| OutL |  |  | 4840158 | 4841363 |
| OutM |  |  | 4841360 | 4841851 |
| OutN |  |  | 4841841 | 4842587 |
| OutO |  |  | 4842697 | 4843548 |
| OutS |  |  | 4846412 | 4846819 |
|  |  |  |  |  |
| **T4SS** |  |  |  |  |
| TraR |  |  | 330706 | 330915 |
| VirB1 |  |  | 331644 | 332309 |
| VirB2 |  |  | 332312 | 332587 |
| VirB4 |  |  | 332597 | 335344 |
| VirB5 |  |  | 335357 | 336073 |
| TraG |  |  | 336084 | 336308 |
| VirB6 |  |  | 336320 | 337348 |
| VirB7 |  |  | 337435 | 337581 |
| VirB8 |  |  | 337568 | 338251 |
| VirB9 |  |  | 338251 | 339141 |
| VirB10 |  |  | 339138 | 340436 |
| VirB11 |  |  | 340426 | 341454 |
| trwh |  |  | 341438 | 341842 |
| VirD4 |  |  | 346963 | 348834 |
| MobC |  |  | 348845 | 349591 |
|  |  |  |  |  |
| **Regulators** |  |  |  |  |
| MarR |  |  | 706701 | 707207 |
| LuxR |  |  | 776968 | 777573 |
| KdgR |  |  | 943588 | 944505 |
| LuxR_2 |  |  | 3180270 | 3180854 |
| VirK |  |  | 3532739 | 3533734 |
| SlyB |  |  | 5249928 | 5250392 |
| RsmA |  |  | 1783946 | 1784131 |
| GacA |  |  | 4608733 | 4610565 |
| LuxS |  |  | 1779589 | 1780104 |
| RpoS |  |  | 1807073 | 1809634 |
| PecT |  |  | 1555091 | 1555867 |
| PecT_1 |  |  | 2161164 | 2162102 |
| PecT_2 |  |  | 3980052 | 3980885 |
| PecT_3 |  |  | 3989798 | 3990700 |
| RsmB |  |  | 3715354 | 3715696 |
|  |  |  |  |  |
| *Rahnella victoriana* BRK18a | |  |  |  |
| **PCWDE** |  |  |  |  |
| CAZy | EC | Enzyme name | Start | End |
| PL5 | 4.2.2.3 | Mannuronate-specific alginate lyase | 904184 | 905518 |
| PL17 | 4.2.2.3 | Mannuronate-specific alginate lyase | 905515 | 907653 |
| PL1 | 4.2.2.2 | Pectate lyase | 406393 | 407490 |
| PL9 | 4.2.2.9 | Pectate disaccharide-lyase | 571850 | 573916 |
| PL22 | 4.2.2.6 | Oligogalacturonate lyase | 601916 | 603097 |
| GH3 | 3.2.1.52 | β-N-acetylhexosaminidase | 254584 | 255612 |
| GH3 | 3.2.1.21 | β-glucosidase | 2882341 | 2884638 |
| GH31 | 3.2.1.177 | α-D-xyloside xylohydrolase | 1366261 | 1368585 |
| GH31 | 3.2.1.20 | α-glucosidase | 2272120 | 2274483 |
| GH31 | 3.2.1.20 | α-glucosidase | 3876798 | 3878834 |
| GH31 | 3.2.1.177 | α-D-xyloside xylohydrolase | 35459 | 37498 |
| GH5 | 3.2.1.8 | Endo-1,4- β-xylanase | 1229845 | 1230924 |
| GH8 | 3.2.1.4 | Cellulase | 3326300 | 3327313 |
| GH43 | 3.2.1.37 | Xylan 1,4-β-xylosidase | 4061547 | 4063106 |
| GH43 | 3.2.1.55 | Non-reducing end α-L-arabinofuranosidase | 4311805 | 4312779 |
| GH4 | 3.2.1.86 | 6-phospho-β-glucosidase | 3250586 | 3251896 |
| GH4 | 3.2.1.86 | 6-phospho-β-glucosidase | 53619 | 54962 |
| GH36 | 3.2.1.22 | α-galactosidase | 3417474 | 3419600 |
| GH1 | 3.2.1.86 | 6-phospho-β-glucosidase | 701275 | 702738 |
| GH1 | 3.2.1.86 | 6-phospho-β-glucosidase | 684078 | 685499 |
| GH1 | 3.2.1.86 | 6-phospho-β-glucosidase | 409139 | 410575 |
| GH1 | 3.2.1.86 | 6-phospho-β-glucosidase | 6762 | 8225 |
| GH1 | 3.2.1.86 | 6-phospho-β-glucosidase | 4063145 | 4064578 |
| GH1 | 3.2.1.86 | 6-phospho-β-glucosidase | 3226343 | 3227725 |
| GH1 | 3.2.1.21 | β-glucosidase | 2561222 | 2562658 |
| GH1 | 3.2.1.86 | 6-phospho-β-glucosidase | 1491576 | 1492973 |
| GH1 | 3.2.1.86 | 6-phospho-β-glucosidase | 1232176 | 1233606 |
| GH2 | 3.2.1.23 | β-galactosidase | 4511548 | 4514646 |
| GH42 | 3.2.1.23 | β-galactosidase | 1584976 | 1587045 |
| GH53 | 3.2.1.89 | Arabinogalactan endo-β-1,4-galactanase | 1587110 | 1588321 |
| CE1 | 3.1.1.1.73 | Feruloyl esterase | 617915 | 619510 |
|  |  |  |  |  |
| **T2SS** |  |  |  |  |
| outB |  |  | 3078302 | 3079822 |
| outC |  |  | 3080023 | 3080856 |
| outD |  |  | 3080840 | 3082819 |
| outE |  |  | 3082823 | 3084346 |
| outF |  |  | 3084346 | 3085563 |
| outG |  |  | 3085576 | 3086031 |
| outH |  |  | 3086034 | 3086555 |
| outI |  |  | 3086555 | 3086944 |
| outJ |  |  | 3086941 | 3087621 |
| outK |  |  | 3087618 | 3088604 |
| outL |  |  | 3088605 | 3089780 |
| outM |  |  | 3089777 | 3090235 |
|  |  |  |  |  |
| **Regulators** |  |  |  |  |
| KdgR |  |  | 349876 | 350667 |
| SlyA |  |  | 424894 | 425328 |
| OmpR |  |  | 3156371 | 3157090 |
| GacA |  |  | 194453 | 195109 |
| VirR |  |  | 597677 | 598084 |
| RsmA |  |  | 2723125 | 2723310 |
| RsmB |  |  | 2517066 | 2517422 |
| HexA |  |  | 2062149 | 2063093 |
| FliA |  |  | 1314825 | 1315547 |
| PhoP |  |  | 277749 | 278423 |
| PhoQ |  |  | 276265 | 277722 |
